# The *Chlamydia* effector CpoS modulates the inclusion microenvironment and restricts the interferon response by acting on Rab35

**DOI:** 10.1128/mbio.03190-22

**Published:** 2023-08-02

**Authors:** Karsten Meier, Lana H. Jachmann, Gözde Türköz, Mohammed Rizwan Babu Sait, Lucía Pérez, Oliver Kepp, Raphael H. Valdivia, Guido Kroemer, Barbara S. Sixt

**Affiliations:** 1 Department of Molecular Biology, Umeå University, Umeå, Sweden; 2 The Laboratory for Molecular Infection Medicine Sweden (MIMS), Umeå University, Umeå, Sweden; 3 Umeå Centre for Microbial Research (UCMR), Umeå University, Umeå, Sweden; 4 Metabolomics and Cell Biology Platforms, Institut Gustave Roussy, Villejuif, France; 5 Centre de Recherche des Cordeliers, Equipe labellisée par la Ligue contre le cancer, Université de Paris, Sorbonne Université, Inserm U1138, Institut Universitaire de France, Paris, France; 6 Department of Molecular Genetics and Microbiology, Duke University School of Medicine, Durham, North Carolina, USA; 7 Department of Biology, Institut du Cancer Paris CARPEM, Hôpital Européen Georges-Pompidou, AP-HP, Paris, France; University of Nebraska Medical Center, Omaha, Nebraska, USA

**Keywords:** intracellular bacteria, cell-autonomous immunity, interferon responses, membrane trafficking, Rab GTPases, membrane microdomains

## Abstract

The obligate intracellular bacterium *Chlamydia trachomatis* inserts a family of inclusion membrane (Inc) proteins into the membrane of its vacuole (the inclusion). The Inc CpoS is a critical suppressor of host cellular immune surveillance, but the underlying mechanism remained elusive. By complementing a *cpoS* mutant with various natural orthologs and variants of CpoS, we linked distinct molecular interactions of CpoS to distinct functions. Unexpectedly, we found CpoS to be essential for the formation of inclusion membrane microdomains that control the spatial organization of multiple Incs involved in signaling and modulation of the host cellular cytoskeleton. While the function of CpoS in microdomains was uncoupled from its role in the suppression of host cellular defenses, we found the ability of CpoS to interact with Rab GTPases to be required not only for the manipulation of membrane trafficking, such as to mediate transport of ceramide-derived lipids (sphingolipids) to the inclusion, but also for the inhibition of Stimulator of interferon genes (STING)-dependent type I interferon responses. Indeed, depletion of Rab35 phenocopied the exacerbated interferon responses observed during infection with CpoS-deficient mutants. Overall, our findings highlight the role of Inc–Inc interactions in shaping the inclusion microenvironment and the modulation of membrane trafficking as a pathogenic immune evasion strategy.

**IMPORTANCE:**

*Chlamydia trachomatis* is a prevalent bacterial pathogen that causes blinding ocular scarring and urogenital infections that can lead to infertility and pregnancy complications. Because *Chlamydia* can only grow within its host cell, boosting the intrinsic defenses of human cells may represent a novel strategy to fight pathogen replication and survival. Hence, CpoS, a *Chlamydia* protein known to block host cellular defenses, or processes regulated by CpoS, could provide new opportunities for therapeutic intervention. By revealing CpoS as a multifunctional virulence factor and by linking its ability to block host cellular immune signaling to the modulation of membrane trafficking, the present work may provide a foundation for such rationale targeting and advances our understanding of how intracellular bacteria can shape and protect their growth niche.

## INTRODUCTION

Intracellular bacteria often thrive within membrane-bound vacuoles that provide a growth niche and protection from host cell-autonomous immune surveillance. The vacuole membrane is heavily modified by bacterial factors that interact with multiple host proteins. Our mechanistic understanding of these interactions remains scarce, which impedes our ability to exploit them as possible targets for therapeutic interventions.

Prominent examples of bacteria that thrive in vacuoles are the obligate intracellular pathogens of the genus *Chlamydia*, which are responsible for a range of diseases in humans and animals (1, 2). For instance, *Chlamydia trachomatis*, a major human pathogen, can cause blinding trachoma (serovars A–C) (3), urogenital infections that entail infertility and pregnancy complications (serovars D–K) (4), as well as the invasive disease lymphogranuloma venereum (serovars L1–L3) (5).

The *Chlamydia* vacuole, termed an inclusion, is established when the elementary body (EB), the infectious non-replicative form of the pathogen, invades a host cell (6). Inside the inclusion, the EB differentiates into the reticulate body (RB), the non-infectious replicative form, which multiplies. Eventually, RBs differentiate back into EBs, which are released by host cell lysis or extrusion of the inclusion. In cultured epithelial cells, *C. trachomatis* typically completes this cycle within 48–72 hours (7).

*Chlamydia* spp. modify the inclusion by secreting a class of membrane-bound effector proteins (8). These inclusion membrane proteins (Incs) interact extensively with host proteins and with each other (9, 10) to mediate a range of processes, such as homotypic inclusion fusion (11), formation of endoplasmic reticulum (ER)–inclusion contact sites (12), cytoskeletal rearrangements (13 - 15), and modulation of host vesicular transport (10, 16, 17).

The Inc CpoS (also known as CTL0481 in *C. trachomatis* serovar L2 and CT229 in serovar D, henceforth referred to as CpoS(L2) and CpoS(D), respectively; Table S1) was identified in a screen for *C. trachomatis* L2 (CTL2) mutants that induced premature death in infected cells (18). CpoS(L2) mutants also induced a robust STING-dependent type I interferon (IFN) response and were impaired in their ability to produce infectious EBs and to survive in the murine upper genital tract (18, 19). Together, these findings revealed CpoS(L2) as a key virulence factor that suppresses cell-autonomous immunity. However, because of the high sequence variability among CpoS orthologs (20, 21), it remained unclear if this function is conserved. Moreover, while both CpoS(L2) and CpoS(D) were found to interact with other Incs (9, 18, 22) and host Rab GTPases (10, 16, 18, 23), the significance of these interactions in suppressing cellular defenses remained unknown.

To better understand how CpoS subverts host cellular defenses, we analyzed the capacity of CpoS variants and orthologs to complement the phenotypes observed in a *cpoS* null mutant and to restore interactions with *Chlamydia* and host proteins. We found that core functions of CpoS are conserved, that CpoS dampens cell death and IFN responses through distinct molecular interactions, and that the inhibition of IFN responses is linked to the ability of CpoS to recruit Rab35 GTPase to the inclusion. Unexpectedly, we also uncovered a role of CpoS in the establishment of inclusion membrane microdomains, highlighting the significance of Inc–Inc associations in shaping *Chlamydia*–host interactions.

## RESULTS

### CpoS orthologs broadly complement a *cpoS* null mutant in *C. trachomatis* L2

Orthologs of CpoS can be found in all serovars of *C. trachomatis*, yet their sequences vary (Fig. S1A) and cluster according to disease tropism (20, 21). CpoS orthologs are also found in the mouse pathogen *Chlamydia muridarum* and the swine pathogen *Chlamydia suis*, but their similarity to CpoS in *C. trachomatis* is relatively low (Fig. S1A). We thus tested if the function of CpoS in blocking cell-autonomous defenses is conserved across serovars and species.

**Fig 1:**
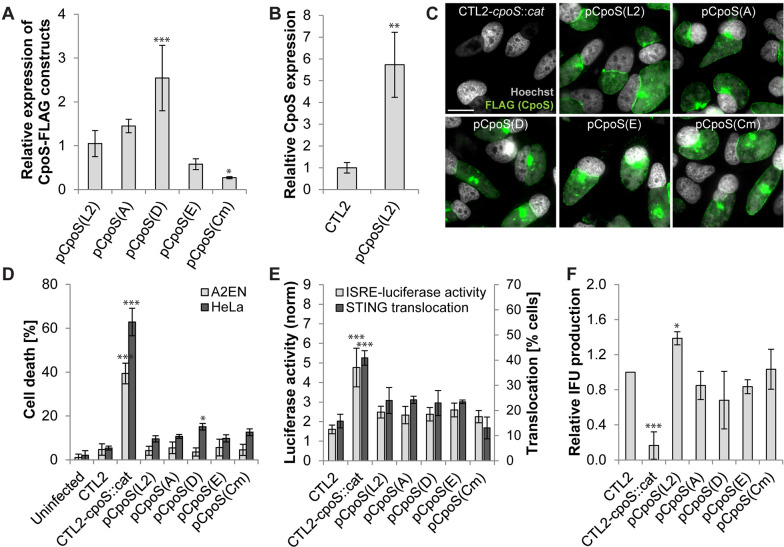
CpoS orthologs broadly complement a *cpoS* null mutant in *C. trachomatis* L2. (**A and B**) Expression levels of CpoS orthologs in infected HeLa cells [10 inclusion forming units (IFU)/cell, 32 hpi] determined by western blot analysis using anti-FLAG (**A**) or anti-CpoS (**B**) antibodies and displayed relative to levels observed during infection with CTL2-*cpoS::cat*/pCpoS(L2)-FLAG (**A**) or CTL2 (**B**). (**C**) Localization of CpoS orthologs in infected HeLa cells (5 IFU/cell, 22 hpi, scale = 20 µm). (**D**) Ability of CpoS orthologs to block cell death in infected cells (10 IFU/cell, 24 hpi). (**E**) Ability of CpoS orthologs to dampen the IFN response. Left *y*-axis: Luciferase activity in infected A2EN-ISRE reporter cells (10 IFU/cell, 14 hpi). Right *y*-axis: Frequency of STING activation (defined as microscopically detectable STING translocation from the ER to perinuclear vesicles) in MLFs (2.5 IFU/cell, 18 hpi, at least 270 cells per condition analyzed). (**F**) Ability of CpoS orthologs to restore the production of IFUs (i.e., EBs) in HeLa cells (measured at 36 hpi and displayed relative to IFU production by CTL2). All quantitative data in Fig. 1 represent mean ± SD [*n* = 6 (A2EN in the panel **D**), *n* = 3 (else); Student’s *t*-test (**B**) or one-way ANOVA (else): indicated are significant differences compared to pCpoS(L2) (**A**) or CTL2 (else)].

We disrupted *cpoS* in CTL2 with an insertion carrying a *cat* resistance gene (Fig. S1B) and then complemented the resulting strain, CTL2-*cpoS::cat*, with plasmids driving the expression of either CpoS(L2) or orthologs derived from other *C. trachomatis* serovars [CpoS(A), CpoS(D), CpoS(E)] or *C. muridarum* [CpoS(Cm)] (Fig. S1A; Table S1 and S2). All orthologs were tagged with a C-terminal FLAG epitope, a modification that does not compromise the function of CpoS(L2) (18). Immunoblots of infected HeLa (human cervical epithelial) cells indicated that the orthologs were expressed at variable amounts (Fig. 1A; Fig. S1C), though always above the endogenous level of CpoS in CTL2 (Fig. 1A and B). Moreover, all orthologs localized correctly to the inclusion membrane in infected cells (Fig. 1C).

Consistent with prior observations made in CpoS-deficient strains (18, 19), infection with CTL2-*cpoS::cat*, but not CTL2*,* induced premature lytic death of HeLa and A2EN (human endocervical epithelial) cells by 24 hours post-infection (hpi) (Fig. 1D). At 14 hpi, we detected an enhanced IFN response as assessed by the expression of a luciferase reporter under the control of the IFN-stimulated response element (ISRE) in an A2EN cell line (Fig. 1E). Moreover, when we infected *Goldenticket* mouse lung fibroblasts (MLFs) that express hemagglutinin (HA)-tagged STING, at 18 hpi, the frequency of cells displaying STING activation (i.e., translocation to post-ER compartments) was increased in cultures infected with CTL2-*cpoS::cat* (Fig. 1E). In addition, CTL2-*cpoS::cat* also exhibited a strongly reduced ability to form infectious EBs (Fig. 1F).

Plasmid-driven complementation of CTL2-*cpoS::cat* with either CpoS(L2) or any of the tested CpoS orthologs abolished or strongly reduced host cell death, blunted the activation of the IFN response, and restored EB production to normal levels (Fig. 1D through F).

Collectively, these findings indicate that CpoS orthologs, despite their variance in amino acid composition, have conserved activities in protecting cells from death, restricting IFN responses, and restoring the full replication potential of *Chlamydia*.

### CpoS variants with mutations in coiled-coil motifs display differential abilities to counteract cell-autonomous defenses

**Fig 2:**
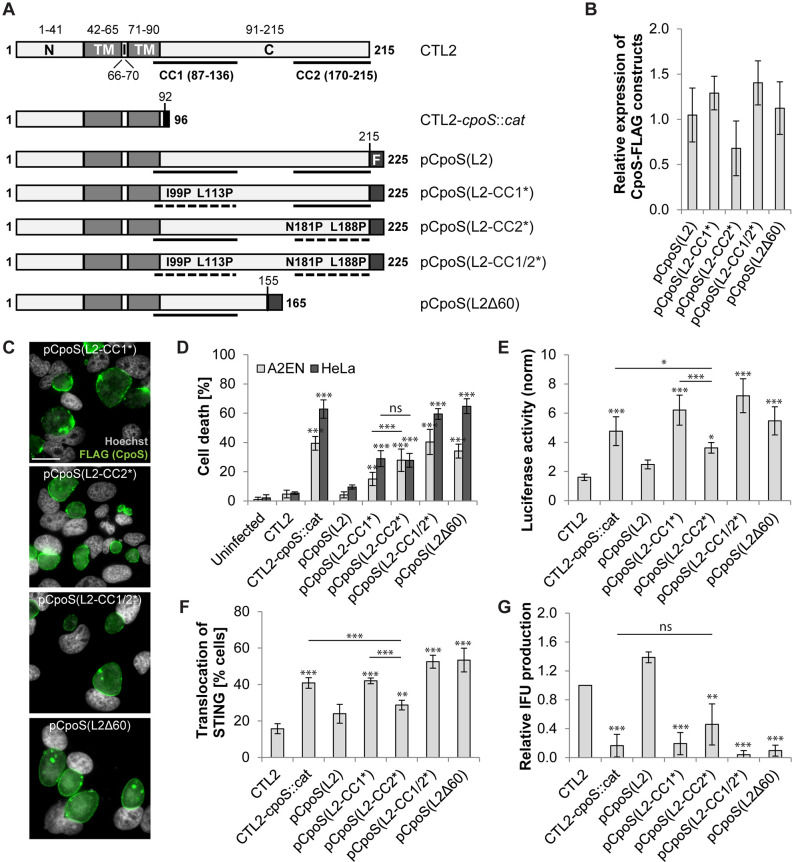
CpoS variants with mutations in coiled-coil motifs display differential abilities to counteract cell-autonomous defenses. (**A**) Predicted domain structure of CpoS(L2) expressed by wild-type, mutant, and complemented strains [N-terminal (N), transmembrane (TM), intra-inclusion (I), and C-terminal (C) domains; coiled-coil (CC) motifs; FLAG (F) tag]. Topology and CC motifs were predicted using Phobius tool (25) and Paircoil2 (26), respectively. Disruptive effects of mutations on CC motifs were predicted using both Paircoil2 (26) and Coils (27). (**B**) Relative expression levels of CpoS variants in infected HeLa cells (10 IFU/cell, 32 hpi) determined by western blot analysis using anti-FLAG antibodies. (**C**) Localization of CpoS variants in infected HeLa cells (5 IFU/cell, 22 hpi, scale = 20 µm). (**D–G**) Ability of CpoS variants to prevent cell death (**D**), block ISRE-driven luciferase expression (**E**), block STING translocation (**F**), and restore IFU (i.e., EB) production (**G**). Details as described in Fig. 1D through F. Note, the experiments were conducted together with those shown in Fig. 1D through F and share the same controls. All quantitative results in Fig. 2 represent mean ± SD [*n* = 6 (A2EN in panel **D**), *n* = 3 (else); one-way ANOVA: indicated are significant differences compared to pCpoS(L2) (**B**) or CTL2 (else, unless indicated otherwise)].

CpoS(L2) has a short N-terminal domain that directs type III secretion (24), a hydrophobic domain with two transmembrane regions that mediate the insertion into the inclusion membrane, and a longer C-terminal domain of unknown function (Fig. 2A). The C-terminal domain is predicted to be exposed to the host cell cytosol during infection and to contain two coiled-coil (CC) motifs (Fig. 2A).

To determine the contribution of the CC motifs to CpoS functions, we transformed CTL2-*cpoS::cat* with plasmids driving the expression of various FLAG-tagged variants of CpoS(L2). These included unmodified CpoS(L2), a truncated CpoS(L2) lacking 60 amino acids (including CC2) at the C-terminus [CpoS(L2Δ60)], and variants in which the α-helices constituting either CC1 or CC2 or both were disrupted by substitutions introducing proline residues [CpoS(L2-CC1*), CpoS(L2-CC2*), and CpoS(L2-CC1/2*)] (Fig. 2A and Table S2). All variants were expressed at comparable levels and localized to the inclusion membrane in infected HeLa cells (Fig. 2B and C; Fig. S1C).

Expression of CpoS(L2Δ60) or CpoS(L2-CC1/2*) in CTL2-*cpoS::cat* failed to protect infected cells from death (Fig. 2D) and resulted in an exaggerated IFN response (Fig. 2E and F). While the expression of CpoS(L2-CC1*) or CpoS(L2-CC2*) partially protected cells from death (Fig. 2D), only CpoS(L2-CC2*) reduced IFN-driven luciferase production and STING activation (Fig. 2E and F). CpoS(L2-CC2*) was also the only modified variant tested that partially restored EB production (Fig. 2G).

Taken together, our findings demonstrate that both suppression of cell death and dampening of IFN production critically rely on the C-terminal domain of CpoS, but through distinct mechanisms. We further reasoned that these mutants could be used to identify the molecular basis of the suppression of IFN responses.

### CpoS deficiency disrupts the formation of inclusion membrane microdomains

*Chlamydia* Incs interact with each other, presumably to form protein interaction networks. CpoS(D) interacts with the Incs CT223/IPAM(D), CT115/IncD(D), and CT222 in bacterial and yeast two-hybrid systems (9, 22). Among these possible interactors, only CTL0476/IPAM(L2) was previously found to interact with CpoS(L2) (18).

**Fig 3:**
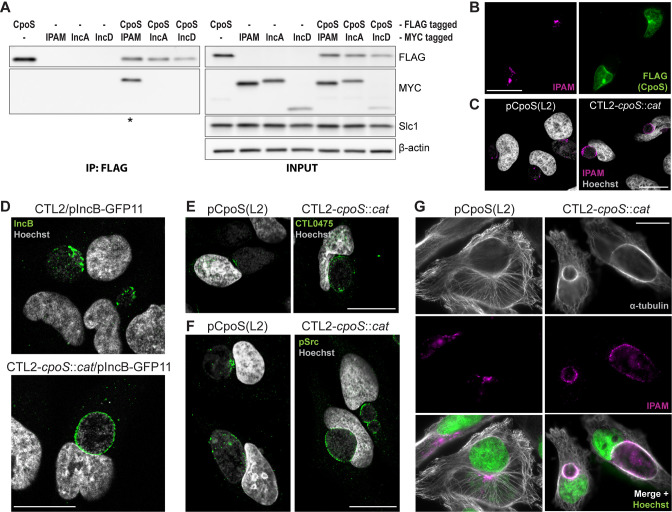
CpoS deficiency disrupts the formation of inclusion membrane microdomains. (**A**) Co-IP confirms the interaction between CpoS and IPAM. HeLa cells were co-infected with CTL2-*cpoS::cat*/pCpoS(L2)-FLAG (2.5 IFU/cell) and CTL2/pIPAM(L2)-MYC, CTL2/pIncA(L2)-MYC, or CTL2/pIncD(L2)-MYC (2.5 IFU/cell), or were infected with each strain individually (5 IFU/cell). CpoS-FLAG was precipitated from lysates at 26 hpi. Samples were analyzed by western blot analysis (* indicates visible Co-IP of MYC-tagged Inc). (**B**) Enrichment of CpoS-FLAG in IPAM-containing microdomains in HeLa cells infected with CTL2-*cpoS::cat*/pCpoS(L2)-FLAG (2.5 IFU/cell, 24 hpi, scale = 40 µm). (**C–F**) Localization of IPAM (**C**), IncB (**D**), CTL0475 (**E**), and active Src kinases (**F**) in infected HeLa cells (5 IFU/cell, 20–24 hpi, scale = 20 µm, confocal). The proteins were detected by immunostaining of endogenous proteins (**C, E, and F**) or by using the split-GFP approach (**D**). (**G**) Architecture of the microtubule cytoskeleton in infected HeLa cells (5 IFU/cell, 22 hpi, scale = 20 µm).

We confirmed the interaction between CpoS(L2) and IPAM(L2) by co-immunoprecipitation (Co-IP) using lysates of HeLa cells that had been co-infected with two strains, one expressing CpoS(L2)-FLAG and the other IPAM(L2)-MYC. Because the inclusions inhabited by the two strains fuse, CpoS(L2)-FLAG and IPAM(L2)-MYC were present on the same inclusion membrane. Thus, when CpoS(L2)-FLAG was precipitated using anti-FLAG beads, immunoblots confirmed Co-IP of IPAM(L2)-MYC (Fig. 3A; Fig. S2A). In contrast, we observed no interactions between CpoS(L2)-FLAG and IncA(L2)-MYC (included as negative control) or between CpoS(L2)-FLAG and IncD(L2)-MYC (considered as possible additional CpoS interactor) (Fig. 3A; Fig. S2A).

IPAM displays a non-uniform distribution at the inclusion membrane with an accumulation at discrete regions termed inclusion membrane microdomains (8, 13, 28). We observed that CpoS was also abundant at these sites (Fig. 3B), yet, in cells infected with CTL2-*cpoS::cat*, IPAM localization was no longer restricted to the microdomains (Fig. 3C). The same was observed for other proteins previously reported to accumulate at inclusion microdomains (29), including IncB, CTL0475 (the CTL2 ortholog of CT222), and active (phosphorylated) host Src kinases (Fig. 3D through F). Of note, CpoS deficiency did not affect the overall expression levels of other Incs, such as IPAM, CTL0475, or IncA (an Inc not enriched in microdomains), or the levels of active Src kinases in infected cells (Fig. S2B through E).

The function of inclusion microdomains is not well understood, yet IPAM has been proposed to modulate the host microtubule (MT) network (13). Accordingly, the loss of CpoS resulted in a modified MT architecture, particularly a more pronounced accumulation of MTs at the periphery of inclusions (Fig. 3G).

In summary, these data demonstrate that CpoS deficiency leads to a general dispersal of inclusion microdomains or prevents their formation, highlighting how the loss of a single Inc can have significant effects on the entire Inc interaction network.

### Disruption of inclusion microdomains is not linked to an augmented cell-autonomous defense

To explore potential links between inclusion microdomains and the subversion of cell-autonomous defenses, we evaluated the capacity of CpoS variants to mediate interactions with IPAM and to restore microdomain formation.

**Fig 4:**
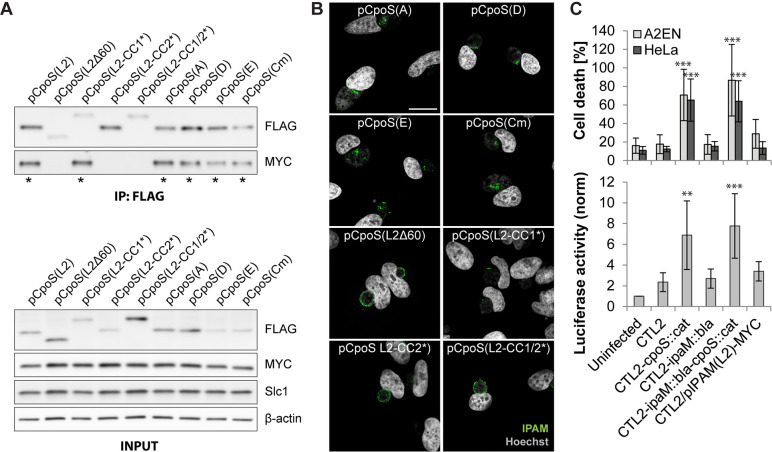
Disruption of inclusion microdomains is not linked to an augmented cell-autonomous defense. (**A**) Co-IP reveals a differential ability of CpoS variants to interact with IPAM. HeLa cells were co-infected with CTL2/pIPAM(L2)-MYC and derivatives of CTL2-*cpoS::cat* expressing variants of CpoS-FLAG (2.5 IFU/cell each). CpoS-FLAG constructs were precipitated from lysates at 26 hpi. Eluates were analyzed by western blot analysis (* indicates visible Co-IP of IPAM(L2)-MYC). (**B**) Localization of IPAM in infected HeLa cells (5 IFU/cell, 20 hpi, scale = 20 µm, confocal). (**C**) *Chlamydia*’s ability to dampen cell death and IFN responses does not depend on IPAM. Luciferase activity in A2EN-ISRE reporter cells (2.5 IFU/cell, 14 hpi) and cell death in A2EN and HeLa cells (2.5 IFU/cell, 24 hpi) (mean ± SD, *n* = 3–7, one-way ANOVA: indicated are significant differences compared to CTL2).

All CpoS orthologs could co-precipitate IPAM(L2)-MYC, yet this interaction was abolished in all CpoS(L2) variants except for CpoS(L2-CC1*) (Fig. 4A). Moreover, microdomain formation was restored by the expression of each of the orthologs and by CpoS(L2-CC1*) but not by the variants that failed to interact with IPAM (Fig. 4B).

Given the differential abilities of CpoS(L2-CC1*) and CpoS(L2-CC2*) to restore proper IPAM localization (Fig. 4B) and to dampen IFN responses (Fig. 2E and F), it is unlikely that IPAM (mis)localization and its consequences on MTs are linked to IFN responses. Indeed, neither a strain deficient for IPAM (Fig. S3A through C) nor the IPAM-MYC-overexpressing strain (Fig. S3B), both of which express CpoS, differed from wild-type CTL2 with respect to host cell killing or induction of IFN responses (Fig. 4C). Furthermore, a CpoS/IPAM double-deficient strain (Fig. S3A through C) behaved like a CpoS single-deficient strain (Fig. 4C).

Interestingly, the more pronounced formation of MT cages seen during infection with CTL2-*cpoS::cat* (Fig. 3G) was independent of IPAM, as it also occurred in cells infected with the CpoS/IPAM double mutant (Fig. S3D). In contrast, IPAM deficiency appeared to abolish the localization of active Src kinases to the inclusion (Fig. S3E), suggesting that deficiency in this Inc also affects inclusion microdomains, though in different ways than observed for CpoS deficiency.

Overall, these observations indicate that the role of CpoS in blocking cell-autonomous defenses is not linked to its role in maintaining inclusion microdomains.

### CpoS mediates the recruitment of Rab GTPases and modulates membrane trafficking

Numerous members of the Rab GTPase family, which influence membrane transport and organelle identity (30), are recruited to the CTL2 inclusion (31). Of note, CpoS(L2) and CpoS(D) interact with multiple Rab proteins (10, 16, 18); and Rab1A (18) and possibly also others (23) are recruited to the inclusion in a CpoS-dependent manner.

**Fig 5:**
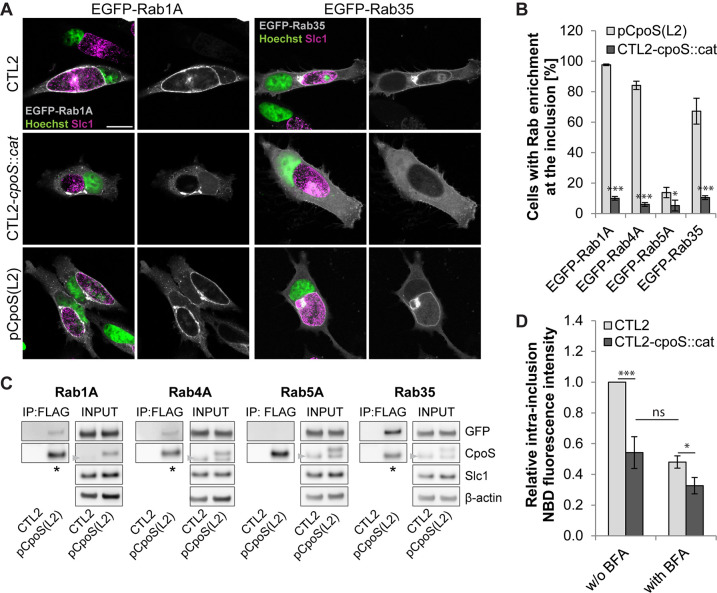
CpoS mediates the recruitment of Rab GTPases and modulates membrane trafficking. (**A**) Localization of EGFP-tagged Rab1A and Rab35 in infected HeLa cells (5 IFU/cell, 24 hpi, confocal, scale = 20 µm). (**B**) Percentage of infected HeLa cells with enrichment of EGFP-Rab proteins at the inclusion (5 IFU/cell, 24 hpi). BFA (3 µg/mL) added at 18 hpi [mean ± SD, *n* = 3 (at least 250 cells per condition), Student’s *t*-test]. (**C**) Co-IP confirms the interaction between CpoS and Rab GTPases. HeLa cells were infected (10 IFU/cell; CTL2 or CTL2-*cpoS::cat*/pCpoS(L2)-FLAG) and transfected with EGFP-Rab expression plasmids. CpoS-FLAG was precipitated from lysates at 26 hpi. Samples were analyzed by western blot analysis (* indicates visible Co-IP of EGFP-tagged Rab proteins). Note that the CpoS-specific antibody detects both endogenous and FLAG-tagged CpoS, yet also a non-specific band [also seen in uninfected cells (not shown)] that overlaps with the band of endogenous CpoS (arrowheads). (**D**) Reduced ceramide acquisition by CpoS-deficient strain. At 14 hpi, infected HeLa cells (5 IFU/cell) were treated with NBD C6-ceramide, in the presence or absence of BFA (3 µg/mL). At 21 hpi, the average intra-inclusion NBD fluorescence intensity was determined and is displayed relative to the average intensity observed in CTL2 inclusions in the absence of BFA (mean ± SD, *n* = 3, one-way ANOVA).

To confirm that CpoS is required for the recruitment of a diverse set of Rab GTPases, we monitored the localization of enhanced green fluorescent protein (EGFP)-Rab fusion proteins in infected HeLa cells. At 24 hpi, Rab1A, Rab4A, and Rab35, but not Rab5A, were prominently enriched at the periphery of CTL2 inclusions (Fig. 5A and B; Fig. S4A). Significantly, the recruitment of Rab1A, Rab4A, and Rab35 to inclusions was absent in cells infected with CTL2-*cpoS::cat* but restored on complementation with CpoS(L2) (Fig. 5A and B; Fig. S4A). Because Golgi mini-stacks accumulate at the periphery of *Chlamydia* inclusions (32), which may complicate the detection of inclusion association for Rab proteins that typically associate with the Golgi (e.g., Rab1A), we quantified Rab recruitment in the presence of brefeldin A (BFA) (Fig. 5B). This drug disassembles Golgi stacks (33, 34) but does not compromise the inclusion association of Rab1A (Fig. S4B). Of note, EGFP-tagged Rab1A, Rab4A, and Rab35, but not Rab5A, also co-precipitated with CpoS(L2)-FLAG from lysates of infected HeLa cells using anti-FLAG beads (Fig. 5C). Conversely, CpoS(L2)-FLAG could be co-precipitated with EGFP-tagged Rab1A, Rab4A, and Rab35 using beads coated with anti-green fluorescent protein (GFP) antibodies (Fig. S4C).

Further microscopic analyses indicated that the recruitment of Rab4B and Rab14 to CTL2 inclusions also depended on CpoS, and while Rab18 was recruited neither to CpoS-containing nor to CpoS-deficient inclusions, recruitment of Rab8A and Rab10 was seen in some cells but occurred independently of CpoS (Fig. S5A). Moreover, the accumulation of Rab6B at the periphery of inclusions was primarily a consequence of the recruitment of Golgi fragments to inclusions, and no difference was seen in the presence or absence of CpoS (Fig. S5A). This latter finding was unexpected, as our Co-IPs detected an interaction between CpoS and Rab6B (Fig. S4C).

The recruitment of Rab proteins has been proposed to reflect the ability of the bacteria to capture host vesicles and hence to acquire lipids (23, 35). In support of this concept, the trafficking of nitrobenzoxadiazole (NBD)-labeled C6-ceramide (or derived labeled lipids) to inclusions was reduced in the absence of CpoS (Fig. 5D). The level of reduction was comparable to that seen when vesicular transport was disturbed by the addition of BFA, though in cells infected with CTL2-*cpoS::cat*, treatment with BFA only caused a minor additional reduction (Fig. 5D). Of note, *C. trachomatis* can acquire host ceramide also via a non-vesicular route involving the recruitment of the ceramide transport protein CERT to inclusion–ER contact sites (12, 36). However, we found recruitment of CERT not to be affected by CpoS deficiency (Fig. S5B).

Taken together, CpoS recruits a diverse set of Rab GTPases to the inclusion and its absence compromises the capacity of *C. trachomatis* to subvert the membrane trafficking machinery of its host cell.

### Rab35 participates in the CpoS-mediated blockade of the IFN response

**Fig 6:**
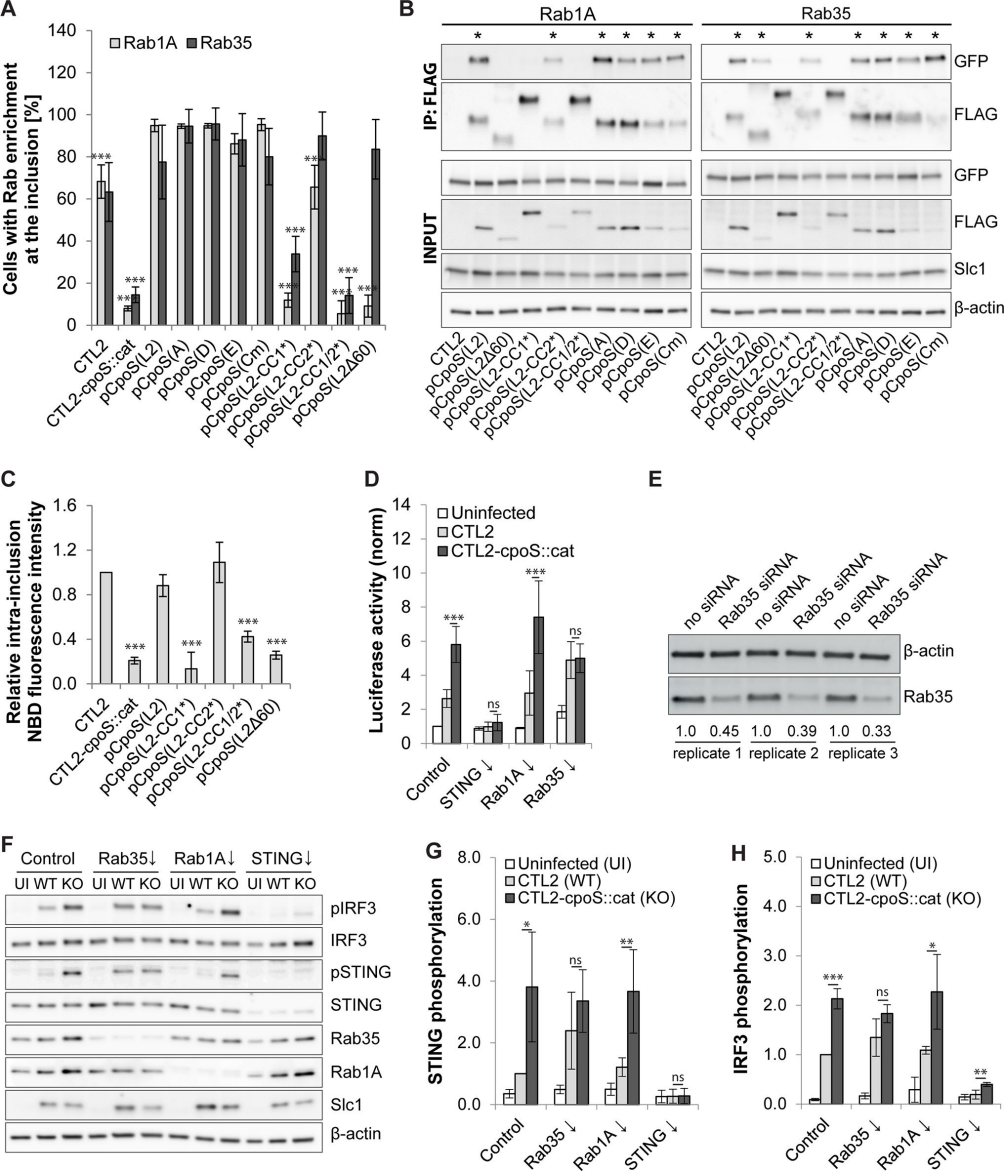
Rab35 participates in the CpoS-mediated blockade of the IFN response. (**A**) Enrichment of EGFP-tagged Rab1A and Rab35 at inclusions (2.5 IFU/cell, 24 hpi). BFA (3 µg/mL) was added at 18 hpi [mean ± SD, *n* = 3 (at least 75 cells per condition), one-way ANOVA: significant differences compared to pCpoS(L2)]. (**B**) Co-IP reveals the differential ability of CpoS variants to interact with EGFP-Rab proteins. CpoS-FLAG variants were precipitated from lysates of infected HeLa cells (5 IFU/cell, 26 hpi). Eluates were analyzed by western blot analysis (* indicates visible Co-IP of EGFP-tagged Rab proteins). (**C**) Differential ability of CpoS variants to restore ceramide acquisition. At 14 hpi, infected HeLa cells (5 IFU/cell) were treated with NBD C6-ceramide. At 21 hpi, the average intra-inclusion NBD fluorescence intensity was determined and is displayed relative to the average intensity observed in CTL2 inclusions (mean ± SD, *n* = 3, one-way ANOVA: significant differences compared to CTL2). (**D**) Effect of siRNA-mediated Rab protein depletion on the inhibition of the IFN response in infected A2EN-ISRE reporter cells. Cells were infected (10 IFU/cell, purified EBs) at 32 hours post-transfection and analyzed at 14 hpi. Displayed are normalized luciferase activities (mean ± SD, *n* = 7, one-way ANOVA). Note, data for an extended set of Rab proteins are displayed in Fig. S7B and C. (**E**) Western blot analysis confirming the depletion of Rab35 in A2EN cells treated with Rab35-specific siRNAs. Protein samples were generated at 46 hours post-transfection in three independent replicates. Relative Rab35 expression levels are indicated below the bands. (**F–H**) The effect of siRNA-mediated Rab35 depletion on STING and IRF3 phosphorylation in infected cells. A2EN cells were infected [5 IFU/cell, purified EBs; UI, uninfected; WT, wild type (CTL2); KO, knockout (CTL2-*cpoS::cat*)] at 36 hours post-transfection, and protein samples were generated at 14 hpi. Shown are western blot images from one representative replicate (**F**) and graphs displaying STING (**G**) and IRF3 (**H**) phosphorylation levels relative to those observed in control cells infected with CTL2 (mean ± SD, *n* = 3, one-way ANOVA).

To explore potential links between Rab recruitment and the subversion of host cell defenses by CpoS, we determined whether our CpoS variants would restore interactions with Rab proteins. When expressed in CTL2-*cpoS::cat*, all the CpoS orthologs restored the recruitment of Rab1A, Rab4A, and Rab35 (Fig. 6A; Fig. S6). Rab recruitment required interactions mediated by CC1, as CpoS(L2-CC1*) and CpoS(L2-CC1/2*) failed to recruit any of the three GTPases, contrasting with CpoS(L2-CC2*), which restored recruitment of all (Fig. 6A; Fig. S6). CpoS(L2Δ60) also lost its ability to recruit Rab1A and Rab4A, while it was still capable of recruiting Rab35 (Fig. 6A; Fig. S6). Co-IP experiments supported these microscopic observations (Fig. 6B). Moreover, consistent with a role for CpoS-Rab interactions in lipid acquisition, CpoS(L2) restored normal levels of ceramide acquisition when expressed in CTL2-*cpoS::cat*, while the mutant variants of CpoS(L2) failed to do so, with the exception of CpoS(L2-CC2*) (Fig. 6C).

These findings, when compared to the differential abilities of mutant CpoS(L2) variants to suppress IFN responses (Fig. 2E and F), indicated that suppression of IFN responses correlates with the ability of CpoS to recruit Rab proteins. Hence, we next evaluated the effect of small interfering RNA (siRNA)-mediated depletion of selected Rab proteins on the IFN response in infected cells. Depletion of individual Rab proteins had variable effects (Fig. S7A and B) but did generally not affect the increase in IFN signaling observed during infection with CTL2-*cpoS::cat* compared to CTL2 (Fig. S7C). A notable exception was the depletion of Rab35, which compromised the ability of CTL2 to dampen luciferase production to a level similar to that induced by infection with CTL2-*cpoS::cat* (Fig. 6D and E; Fig. S7B and C). Hence, Rab35 depletion phenocopied the effects of CpoS deficiency as it relates to enhanced IFN responses. A similar effect was seen when we assessed more directly the activation of the STING–TBK1–IRF3 signaling pathway in infected cells by monitoring the phosphorylation of STING and IRF3 by western blot analysis (Fig. 6F through H). Of note, depletion of Rab35 did not generally enhance STING signaling, as no enhancement of STING or IRF3 phosphorylation was seen in uninfected cells or in cells transfected with DNA (Fig. S7D through F), pointing to a specific role of Rab35 in modulating immune signaling during infection.

In an attempt to determine how Rab35 inhibits the IFN response during *Chlamydia* infections, we evaluated possible roles for previously described Rab35 effectors (37). We observed that the effector ACAP2 was not recruited to the inclusion, while OCRL1 and FSCN1 were recruited, but independently of CpoS (Fig. S8A). Interestingly, MICAL1 and MICALL1 were recruited to the inclusion in a manner dependent on both CpoS and Rab35 (Fig. S8A). Nonetheless, siRNA-mediated depletion of neither of these Rab35 effectors exacerbated ISRE-driven luciferase expression or STING phosphorylation in cells infected with CTL2 (Fig. S8B through D). It is possible that relevant Rab35 effectors linking CpoS–Rab35 interaction to the subversion of IFN responses remain to be identified.

Taken together, CC1 and the far C-terminus of CpoS mediate interactions with Rab GTPases, and the CpoS–Rab35 interaction contributes to the CpoS-mediated inhibition of IFN responses to *Chlamydia* infections.

## DISCUSSION

**Fig 7:**
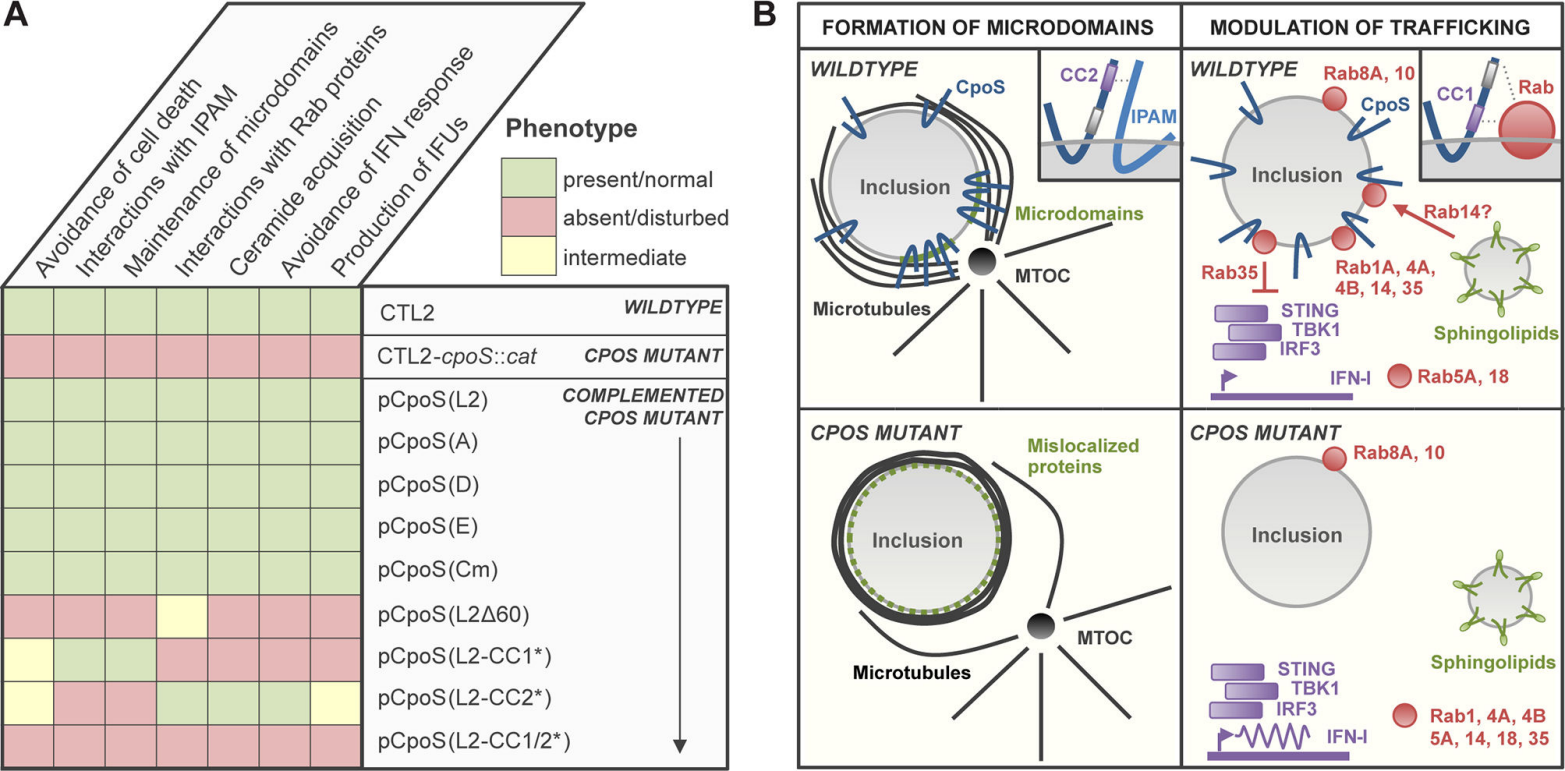
Graphical summary of discoveries reported in this study. (**A**) Overview of the phenotypes of the *cpoS* mutant and the capabilities of distinct CpoS homologs and variants to complement the observed defects. (**B**) Illustration summarizing our two main findings, that is, a role of CpoS in the maintenance of inclusion microdomains (left), and a role of CpoS-Rab35 GTPase interactions in the suppression of the host cellular type I IFN response (right). In brief, we showed that CpoS itself is enriched at inclusion microdomains and that its absence disrupts the microdomain localization of multiple other inclusion-associated proteins, as well as the cytoskeletal architecture at the inclusion. At the molecular level, this function of CpoS in microdomains depends on an intact CC2 motif in CpoS, a motif also required for CpoS–IPAM interactions, strongly suggesting a role for IncInc interactions in the maintenance of inclusion microdomains. In addition, we found that a functional CC1 motif in CpoS and in part also additional amino acids at the C-terminus of CpoS are required for CpoS to interact with host Rab GTPases and to recruit these proteins to the inclusion. Significantly, CpoS–Rab interactions not only play a role in the modulation of host membrane trafficking, for example, sphingolipid transport to inclusions, but also in the suppression of the host cellular type I IFN response. Specifically, we revealed a role for Rab35 in the dampening of STING/TBK1/IRF3 signaling in cells infected with *C. trachomatis*.

In this study, we characterized the interaction partners and biological functions of orthologs and modified variants of CpoS (Fig. 7A) to determine the mechanism by which CpoS subverts host cell defenses. We made two key discoveries (Fig. 7B): (i) CpoS is essential for the proper formation and/or maintenance of inclusion microdomains. Its absence severely disrupts the localization of multiple inclusion-associated proteins, thus significantly altering the microenvironment of the *Chlamydia* vacuole (Fig. 3C through G). (ii) CpoS suppresses host type I IFN responses independently of its function in microdomain formation (Fig. 2E and F; Fig. 4B). Instead, it modulates IFN signaling by modulating membrane trafficking via its interactions with Rab proteins (Fig. 2E and F; and Fig. 6A), more specifically via acting on Rab35 (Fig. 6D through H). In addition, we also established that the core functions and interactions of CpoS are conserved among *Chlamydia* biovars and species (Fig. 1D through F; Fig. 4A and B; Fig. 6A and B; Fig. S6).

The selective recruitment of Rab proteins to the *Chlamydia* inclusion is an established phenomenon (31), but what role CpoS plays in this process has only recently been demonstrated genetically (18, 23). We confirmed CpoS as a broad recruiter of Rab proteins (Fig. 5A and B; Fig. S4A and B; Fig. S5A) and determined that CpoS–Rab interactions require an intact CC1 region (Fig. 6A and B; Fig. S6). Moreover, our observations with the CpoS(L2Δ60) variant indicate that interactions with some Rab proteins additionally depend on residues located at the C-terminus of CpoS (Fig. 6A and B; Fig. S6). Overall, this is consistent with reports regarding the implication of residues 120 (in CC1) and 198–215 (at the far C-terminus) in CpoS–Rab interactions (16, 23). However, in contrast with prior reports (23, 31, 38), we did not find a clear CpoS dependence for the recruitment of Rab6, Rab8A, and Rab10 (Fig. S5A).

Modulation of Rab localization or activity allows intracellular bacteria to control the trafficking of their vacuole and its interactions with host cellular compartments (39). Endocytic Rab proteins are excluded from the *Chlamydia* inclusion presumably to avoid lysosomal targeting of the bacteria (31). Others may be recruited to enable the capture of vesicles that contain lipids important for bacterial replication (35). Indeed, depletion or inhibition of certain Rab proteins, including Rab14, which we found to be recruited by CpoS (Fig. S5A), decreases ceramide (sphingolipid) transport to inclusions and the generation of infectious EBs (40 - 42). Consistently, we found the acquisition of ceramide (-derived lipids) by inclusions to be reduced in the absence of CpoS (Fig. 5D), confirming the functional significance of CpoS–Rab interactions.

Significantly, our work revealed that only those variants of CpoS that recruited Rab proteins were capable of dampening STING activation and ISRE-driven gene expression (Fig. 2E and F; Fig. 6A and B; Fig. S6). Moreover, we identified a specific role for Rab35, a protein engaged in endocytic recycling (43), in the CpoS-mediated inhibition of the STING-dependent IFN response (Fig. 6D through H). This is interesting because various intracellular bacteria modulate Rab35 activity or localization (44 - 48), implying that the suppression of the IFN response through the exploitation of Rab35 function could be a more widespread pathogenic strategy.

Since CpoS(L2Δ60) restores Rab35 recruitment (Fig. 6A; Fig. S6), but not the inhibition of the IFN response (Fig. 2E and F), we predict that Rab35 needs to act with additional factors recruited by CpoS, potentially other Rab proteins, to dampen STING activation. We failed to observe similar IFN response-exacerbating effects after individual depletion of additional Rab proteins (Fig. S7A through C). However, we cannot exclude that we could have identified such effects, if we had extended our analyses to a larger set of Rab proteins, if we had depleted more than one Rab protein at once, or if we had achieved better knockdown efficiencies.

Interestingly, we did not observe strong evidence for a connection between CpoS–Rab interactions and the inhibition of premature death that occurs in cells infected with *cpoS* mutants (Fig. 2D; Fig. 6A and B; Fig. S6). This is consistent with our previous finding that the cell death response does not require a functional IFN response (18). Restoring Rab recruitment and inhibition of the IFN response, by expression of CpoS(L2-CC2*), also only marginally improved the formation of infectious EBs (Fig. 2G), suggesting that the disruption of EB generation in the absence of CpoS is primarily caused by the premature death of the host cell. However, it is plausible that the inhibition of the IFN response has implications for the pathogenicity of *in vivo* infections, in which the subversion of non-cell-autonomous immune responses involving specialized immune cells should increase the virulence of *Chlamydia*.

Our additional finding that CpoS regulates the formation and/or maintenance of inclusion microdomains was unexpected, as CpoS had not previously been reported to associate with these structures. Inclusion microdomains have been proposed to act as hubs that regulate the trafficking of inclusions to, and their stable association with, centrosomes (49, 50), the MT architecture (13), and bacterial exit by extrusion (51, 52). Likely, many more functions remain to be discovered. Hence, the mislocalization of multiple host and bacterial microdomain-associated proteins in cells infected with *cpoS* mutants is expected to compromise numerous interactions between the host and inclusions. These far-reaching effects seen after the disruption of a single bacterial effector also remind us to be cautious when discerning the role of individual virulence factors.

Of note, the overexpression of certain Incs can affect the abundance of other Incs (53) and the overexpression of microdomain-associated Incs can disrupt their microdomain localization (54). However, in the present study, we did not observe changes in the protein levels of the microdomain Incs IPAM and CTL0475 as a consequence of CpoS deficiency or overexpression of CpoS in the complemented *cpoS* mutant (Fig. S2B and C). Interestingly, a prior study reported that IncA deficiency also disrupts the localization of IPAM to inclusion microdomains (55), though it remains elusive if this is accompanied by broader effects on microdomains and/or changes in IPAM abundance.

Altogether, this work highlights the importance of Inc–Inc associations in shaping the microenvironment of the *Chlamydia* inclusion, as well as the importance of CpoS–Rab interactions for the suppression of the IFN response by *C. trachomatis*. These mechanistic insights may support future efforts to explore and exploit CpoS-mediated processes as potential therapeutic targets.

## MATERIALS AND METHODS

An extended description of methods can be found in the supplemental material (Methods S1).

### Cell culture

HeLa cells (ATCC CCL-2), Vero cells (ATCC CCL-81), *Gt* MLFs expressing STING-HA (56), A2EN cells (57), and A2EN-ISRE reporter cells (18) were maintained under standard cell culture conditions.

### Transient expression in human cells

Plasmids enabling the expression of EGFP-tagged human Rab proteins (31, 47) or cytosolic GFP1-10 (58) were provided by Marci Scidmore and Kevin Hybiske, respectively. Plasmids enabling the expression of HA-tagged Rab35 effector proteins were generated in this study, as described in Methods S1. Plasmid p2TK2-SW2 (59) was used to stimulate the STING pathway in uninfected cells. Cells were transfected using jetPRIME (Polyplus) or Lipofectamine 2000 (Invitrogen).

### *Chlamydia* strains and infection

Infection inocula (crude bacterial preparations and density gradient purified EBs) of *C. trachomatis* L2/434/Bu (CTL2, ATCC VR-902B) or derivative strains generated in this study (Table S2) were prepared as described in Methods S1. Cells were infected by the addition of bacteria [inclusion-forming unit (IFU)/cell as specified], followed by centrifugation (1,500× *g*, 30 minutes, 23°C).

### Gene disruption in *Chlamydia*

CTL0476 (*ipaM*) was disrupted in CTL2 by TargeTron using retargeted (Table S3) pDFTT3 (60). CTL0481 (*cpoS*) was disrupted using a derivative of pDFTT3, which was retargeted toward CTL0481 (18) and modified [as described previously (61)] to contain a *cat* selection marker in the intron. Bacteria were transformed using the CaCl_2_ approach (62), selected in the presence of penicillin G (Merck) or chloramphenicol (Merck), and plaque-purified (18). Intron insertion at target sites was verified by PCR (Table S3) and sequencing (Eton Bioscience).

### Gene expression in *Chlamydia*

For the expression of FLAG-tagged CpoS proteins, the tag was inserted between the KpnI and SalI sites of p2TK2-SW2 (59). DNA fragments coding for CpoS proteins and their promoters were amplified (Table S3) or obtained as synthetic gene blocks (IDT) and then inserted between the AgeI and KpnI sites of the vector. For the expression of MYC-tagged Incs, the Inc genes and their promoters were amplified (Table S3) and then inserted between the AgeI and NheI sites of vector p2TK2-SW2 (59). Plasmids enabling the expression of mCherry (59) or inducible expression of GFP11-tagged IncB (CT232) (58) were gifts from Isabelle Derré and Kevin Hybiske, respectively. Bacteria were transformed as described above. Expression of GFP11-tagged IncB was induced by the addition of anhydrotetracycline (Clontech).

### Quantification of cell death and IFN responses

LDH activity in culture supernatants was quantified using the photometric *in vitro* cytotoxicity kit (Merck). Activity detected in cell-free medium was subtracted, and values were normalized to activity detected in a total cell lysate to calculate the percentage of dead cells. The induction of type I IFN-dependent genes was assessed using an A2EN-ISRE-luciferase reporter cell line as previously described (18). Luminescence values were normalized to the mean luminescence detected in uninfected (non-siRNA-treated) wells.

### Determination of infectious progeny

Infectious progeny was determined as previously described (18). In brief, confluent monolayers of HeLa cells were infected using a low infection dose. At 36 hpi, cell lysates were prepared by H_2_O-based cell lysis. IFUs in the initial inoculum (input) and collected cell lysates (output) were quantified by infecting confluent monolayers of Vero cells with serial dilutions, followed by the fluorescence microscopic determination of inclusion numbers at 28 hpi. From the IFUs detected in the input and the output, the number of IFUs formed per infected cell was determined and then normalized to the yields observed for CTL2.

### Ceramide (sphingolipid) acquisition

At 14 hpi, cells were incubated for 15 minutes at 4°C, rinsed with cold Hanks' Balanced Salt Solution (HBSS; Gibco), and then incubated for 30 minutes at 4°C in HBSS containing 5 µM bovine serum albumin (BSA)-complexed NBD C6-ceramide (Thermo Fisher Scientific). Subsequently, the cells were rinsed twice, incubated for 6 hours in medium (37°C, 5% CO_2_), stained with Hoechst 33342 (2 µg/mL, 10 minutes), washed twice, and imaged live (ImageXpress Micro XL; Molecular Devices) at about 21 hpi. BFA (3 µg/mL) was added to some wells at 12 hpi and was also present during the incubation with ceramide. Inclusions were detected in MetaXpress (Molecular Devices), and the average intra-inclusion NBD fluorescence intensity was determined and normalized to the average intensity observed during infection with CTL2.

### siRNA-mediated gene silencing

Cells were transfected with siRNAs (Dharmacon; Table S4) using DharmaFECT-1 (Dharmacon). In control transfections, siRNA buffer (Dharmacon) was added instead of siRNAs.

### Fluorescence microscopy

Cells were fixed, permeabilized, and blocked, as described previously (18). Subsequently, the cells were incubated with primary antibodies (Table S5), washed with Dulbecco’s phosphate-buffered saline (DPBS; Gibco), incubated with Hoechst 33258/33342 (Invitrogen; 2–10 µg/mL) and Alexa Fluor (488, 555, or 647)-labeled secondary antibodies (Invitrogen; 1:250–1:1,000), and then washed again with DPBS. Cells grown and stained on glass coverslips were transferred to microscope slides and embedded in Mowiol (18) or ProLong Glass Mountant (Invitrogen). Images were taken with epifluorescence microscopes (Zeiss Axio Observer.Z1, Zeiss Axio Imager.Z2), confocal microscopes (Zeiss LSM 780, Leica SP8), and high-content imaging platforms [ImageXpress Micro XL (Molecular Devices) and Cellomics ArrayScan VTI (Thermo Fisher Scientific)]. The percentage of cells displaying STING translocation, recruitment of EGFP-Rab fusion proteins, or recruitment of HA-tagged Rab effector proteins was determined by manual inspection of microscopic images.

### Co-immunoprecipitation

Cells were lysed in Pierce IP lysis buffer (Thermo Fisher Scientific). Lysates were then homogenized (0.33 × 12-mm needle) and cleared by centrifugation (17,000–20,000× *g*, 4°C, 20 minutes). For IP of FLAG-tagged proteins, ANTI-FLAG M2 magnetic beads (Merck) were incubated with the lysate and then washed with lysis buffer. Proteins were eluted by boiling (3 minutes, 100°C) in Laemmli buffer or by incubation in 0.1 M glycine/HCl (pH 3.0) followed by neutralization. For IP of EGFP fusion proteins, GFP-Trap_MA beads (ChromoTek) were incubated with lysate and then washed with Tris-buffered saline (TBS)/EDTA buffer. Proteins were eluted by boiling. Aliquots from the initial lysate (lysate fraction, also known as input), lysate after incubation with beads (unbound fraction), and the buffer used for the last wash step (wash fraction) were also collected.

### Western blot analysis

Protein extracts were prepared by cell lysis in boiling 1% SDS buffer (18). Protein extracts (and Co-IP samples) were then mixed with loading buffer and denatured (10 minutes, 95°C–100°C), before separation by SDS-PAGE and transfer to nitrocellulose membranes (Bio-Rad). Membranes were blocked [5% milk or 3% BSA in Tris-buffered saline with 0.1% Tween 20 (TBST)], incubated with primary antibodies (Table S5), washed with TBST, incubated with horseradish peroxidase (HRP)-conjugated secondary antibodies (Southern Biotech or Thermo Fisher Scientific, 1:10,000–1:50,000), and then washed again with TBST. Membranes were developed using HRP substrate [ECL Prime (GE Healthcare) or SuperSignal West Pico PLUS (Thermo Fisher Scientific)]. Membranes were stripped with Restore Plus (Thermo Fisher Scientific) and blocked before the detection of additional targets. Band intensities were quantified using Image Quant TL (GE Healthcare). Expression levels of bacterial and host proteins were normalized to the expression of the *Chlamydia* protein Slc1 and host β-actin, respectively.

### Statistical analysis

Statistical analysis was conducted in GraphPad Prism 8. The following statistical tests were used when indicated in the figure legends: one-way analysis of variance (ANOVA; with Newman–Keuls *post hoc* test) and Student’s *t*-test (unpaired two-tailed, assuming equal variance). The following significance levels were considered: *P < 0.05; **P < 0.01; ***P < 0.001; ns, not significant.

## SUPPLEMENTAL MATERIAL

Methods S1 mbio.03190-22-s0001.pdf Extended description of materials and methods.

Figure S1 mbio.03190-22-s0002.tif Conservation of *cpoS*, insertional inactivation of *cpoS*, and complementation with plasmid-expressed FLAG-tagged CpoS orthologs and variants.

Figure S2 mbio.03190-22-s0003.tif Localization of MYC-tagged Incs and effects of CpoS-deficiency on Inc and active Src levels.

Figure S3 mbio.03190-22-s0004.tif Insertional inactivation of *ipaM*, and effects of IPAM-deficiency on microtubule architecture and recruitment of active Src kinases.

Figure S4 mbio.03190-22-s0005.tif CpoS mediates recruitment of a diverse set of Rab proteins.

Figure S5 mbio.03190-22-s0006.tif CpoS dependence or independence of the recruitment of selected Rab proteins and the ceramide transfer protein CERT to the inclusion.

Figure S6 mbio.03190-22-s0007.tif Differential abilities of CpoS orthologs and variants to mediate Rab protein recruitment to inclusions.

Figure S7 mbio.03190-22-s0008.tif Effects of Rab protein depletion on the IFN response.

Figure S8 mbio.03190-22-s0009.tif Effects of Rab35 effector depletion on the IFN response.

Supplemental Tables mbio.03190-22-s0010.pdf Tables S1-S5.

ASM does not own the copyrights to Supplemental Material that may be linked to, or accessed through, an article. The authors have granted ASM a non-exclusive, world-wide license to publish the Supplemental Material files. Please contact the corresponding author directly for reuse.
