# Supplementary material for: The Chlamydia effector CpoS modulates the inclusion microenvironment and restricts the interferon response by acting on Rab35
Source: mBio. 2023 Aug 2;14(4):e03190-22. doi: 10.1128/mbio.03190-22 (PMC10470785; doi:10.1128/mbio.03190-22)
Supplement: Methods S1 — Extended description of materials and methods. [file mbio.03190-22-s0001.pdf]

Methods S1. Extended description of materials and methods

Cell culture

HeLa cells (ATCC CCL-2), Vero cells (ATCC CCL-81), and *Goldenticket* mouse lung fibroblasts expressing STING-HA (1) were grown in Dulbecco's Modified Eagle's Medium (Gibco) supplemented with 10% heat-inactivated fetal bovine serum (Gibco). In selected experiments, *i.e.* such including an analysis of cell death or the imaging of live cells, a DMEM medium devoid of phenol red was used to avoid interference with measurements of absorbance or fluorescence. A2EN (2) and A2EN-ISRE reporter cells (3) were cultivated in Keratinocyte-SFM (Gibco) supplemented with 10% heat-inactivated fetal bovine serum (Gibco). Cultures were maintained in a humidified incubator (37°C, 5% CO_2) and routinely tested for *Mycoplasma* contamination using a PCR assay described elsewhere (4) or by using a commercial PCR-based *Mycoplasma* detection assay (VenorGeM, Minerva).

Transient expression in human cells

Plasmids enabling expression of EGFP-tagged human Rab proteins were kindly provided by Marci Scidmore (formerly at Cornell University). The generation of these plasmids, which are derivatives of pEGFPC1 and pEGFPC2 (Clontech), was described in previous publications (5, 6). To confirm the identity of the cloned Rab genes, plasmid inserts were sequenced (Eurofins or GATC sequencing services) using primer pEGFPC1for (Table S3) and the sequences were compared with respective mRNA sequences available at NCBI gene (7). Plasmid pcDNA3.1-Cyto-GFP1-10 (8), enabling expression of cytosolic GFP1-10 for the detection of GFP11-tagged IncB, was kindly provided by Kevin Hybiske (University of Washington). Plasmids enabling expression of HA-tagged Rab35 effector proteins were generated as follows: Sequences encoding human MICAL1 (NM_001286613.2), MICALL1 (NM_033386.4), or FSCN1 (NM_003088.4) with C-terminal HA tag (flanked by HindIII and NotI restriction sites) were purchased as gene blocks (ThermoFisher or IDT), digested with HindIII and NotI, and inserted into HindIII/NotI-digested vector pcDNA3.1. Sequences encoding human OCRL (NM_000276.4) and ACAP2 (NM_012287.6) were PCR-amplified from cDNA (generated with ProtoScript II First Strand cDNA Synthesis Kit, NEB) using primers introducing a C-terminal HA-tag and BamHI and NotI restriction sites (Table S3). PCR products were then digested and inserted into BamHI/NotI-digested vector pcDNA3.1. Plasmid inserts were confirmed by sequencing (GATC sequencing services). Plasmid p2TK2-SW2 (9) was used to stimulate the STING pathway in uninfected cells. Cells were transfected either 2-5 hours before infection with *Chlamydia* or at 1 hour post

infection (hpi) using jetPRIME transfection reagent (Polyplus) or lipofectamine 2000 (Invitrogen) with a medium exchange after 2-4 hours.

Chlamydia strains and infection

Experiments were carried out with *C. trachomatis* strain L2/434/Bu (CTL2, ATCC VR-902B) and the derivatives generated in this study (Table S2). Two different procedures were used to prepare infection inocula. First, to prepare crude bacterial preparations, bacteria were propagated in Vero cells and harvested at about 36-48 hpi by H_2O-mediated lysis of host cells. This was followed by addition of 1/4 volume of 5X SPG (sucrose-phosphate-glutamate) buffer (375 g/l sucrose, 2.5 g/l KH_2PO_4, 6 g/l Na_2HPO_4, 3.6 g/l glutamic acid, pH 7.5), centrifugation at low speed (500 x g, 10 min, 4°C) to remove host cell debris, brief sonication, and centrifugation at higher speed (17,000 x g, 10 min, 4°C) to pellet bacteria. Bacteria were then resuspended in 1X SPG buffer, briefly sonicated, and stored at -80°C. Second, for selected experiments, EBs present in crude bacterial preparations were further purified by density gradient centrifugation. In brief, we first generated O95NaCl, *i.e.* a 95% dilution of omnipaque 350 (780 mOsmol/kg, GE Healthcare) supplemented with additional 211.25 mM NaCl for osmolality adjustment. Bacteria in SPG buffer were then sonicated and layered on top of 8 ml of a 30% dilution of O95NaCl (diluted in SPG), followed by ultracentrifugation (40,000 x g, 40 min, 4°C). Subsequently, the bacteria found in the pellet were resuspended in 1X SPG buffer, sonicated, and layered on top of a gradient consisting of 3 ml of 54% O95NaCl, 5 ml of 44% O95NaCl, and 2 ml of 40% O95NaCl (O95NaCl dilutions in SPG), followed by ultracentrifugation (40,000 x g, 1 h, 4°C). Purified EBs were then collected from the 44%/54% interface, diluted in 1X SPG buffer, and pelleted by centrifugation (30,000 x g, 30 min, 4°C). Subsequently, the bacteria were resuspended in 1X SPG buffer, again briefly sonicated, and stored at -80°C. Bacterial preparations prepared by either of the two approaches were shown to be free of *Mycoplasma* contamination by PCR, as described above for cell lines. To determine the number of infectious bacteria (*i.e.* the inclusion-forming units (IFUs)) contained in the preparations, monolayers of Vero cells in 96-well plates were infected with serial dilutions of the bacteria. At 28 hpi, cells were fixed with 4% formaldehyde and inclusions were stained with antibodies targeting the chlamydial protein Slc1 (procedure described below). Inclusions were counted using a Cellomics ArrayScan VTI HCS automated imaging system (ThermoFisher). To conduct infections, cells were seeded in multi-well plates, followed by addition of bacteria (number of IFUs/cell as specified), centrifugation (1500 x g, 30 min, 23°C), and incubation (37°C, 5%CO_2) for the indicated periods of time.

Gene disruption in *Chlamydia*

CTL0476 (*ipaM*) was disrupted in CTL2 via the TargeTron approach (10). The primers IBS-CTL0476, EBS1d-CTL0476, and EBS2-CTL0476 (Table S3), as well as EBS universal (Merck), were used to retarget vector pDFTT3 (10) for this purpose. CTL0481 (*cpoS)* was disrupted using a derivative of pDFTT3, which was retargeted towards CTL0481 (3) and modified (as described previously (11)) to contain a *cat* (chloramphenicol resistance) gene instead of a *bla* (β-lactamase resistance) gene in the intron. Bacteria were transformed using the $CaCl_2$ approach (12) and selected in presence of 1 U/ml penicillin G (Merck) or 0.5 µg/ml chloramphenicol (Merck), first added at 12 hpi. Bacteria were then plaque-purified (3) in presence of 5 U/ml penicillin G or 1 µg/ml chloramphenicol. Intron insertion at correct target sites was verified by PCR (using primers shown in Table S3) and sequencing of the resulting PCR products (Eton Bioscience).

Gene expression in *Chlamydia*

To enable expression of CpoS orthologs and variants with a C-terminal FLAG tag, a DNA fragment encoding a FLAG tag and a stop codon was inserted between the KpnI and SalI restriction sites of vector p2TK2-SW2 (9). DNA fragments coding for CpoS proteins (without stop codon) and their promoters (210 bp sequence upstream of the start codon) were then PCR-amplified from genomic DNA (using primers shown in Table S3), or obtained as synthetic gene blocks (IDT), and inserted between the AgeI and KpnI restriction sites of the vector. To enable expression of MYC-tagged Incs, the genes encoding the Incs (CTL0476 (*ipaM*), CTL0374 (*incA*), CTL0370 (*incD*)) and their promoters (210 bp sequence upstream of the start codon) were PCR-amplified (using primers shown in Table S3), digested with AgeI (or SgrAI in case of CTL0476) and NheI, and inserted into AgeI/NheI digested p2TK2-SW2 (9). Vector p2TK2-SW2-mCherry (9), enabling constitutive expression of mCherry, was a kind gift from Isabelle Derré (University of Virginia). Vector pTL2-tetO-IncB-GFP11x7-flag (8), enabling inducible expression of IncB (CT232) tagged at its C-terminus with seven copies of GFP11 (RDHMVLHEYVNAAGIT) and one copy of FLAG (DYKDDDDK), was a kind gift from Kevin Hybiske (University of Washington). Bacteria were transformed as described above. Expression of IncB-GFP11x7-FLAG was induced by addition of 4 ng/ml anhydrotetracycline (Clontech) at 0 hpi (*i.e.* after the centrifugation step that followed addition of the bacteria).

Quantification of cell death and IFN responses

LDH activity in culture supernatants, an indicator for host cell lysis, was quantified using the photometric *in vitro* cytotoxicity kit (Merck), according to the manufacturer's instructions. Activity

detected in cell-free medium was subtracted and values were normalized to activity detected in a total cell lysate to calculate the percentage of dead cells. The induction of type I IFN-dependent genes was assessed using an A2EN-ISRE-luciferase reporter cell line as previously described (3). Cells were lysed by addition of a 1:1 mixture of Hanks' Balanced Salt Solution (HBSS; Gibco) and Britelite plus reagent (PerkinElmer), before measurement of luminescence. Luminescence values were normalized to the mean luminescence detected in uninfected (non-siRNA-treated) wells. Absorbance and luminescence measurements were made using an EnSpire 2300 (PerkinElmer), SpectraMax i3 (Molecular Devices) or Infinite 200 (Tecan) plate reader. The same instrument was used for all replicates of the same experiment.

Determination of infectious progeny

Infectious progeny was determined as previously described (3). In brief, confluent monolayers of HeLa cells in 96-well plates were infected using a low infection dose (< 50% infected cells). At 36 hpi, cell lysates were prepared by H_2O-based cell lysis. IFUs in the initial inoculum (input) and collected cell lysates (output) were quantified by infecting confluent monolayers of Vero cells with serial dilutions, followed by the fluorescence microscopic determination of inclusion numbers at 28 hpi (as described above for the titering of bacterial stocks). From the IFUs detected in the input and the output, the number of IFUs formed per infected cell was determined and then normalized to the IFU production observed for CTL2.

Ceramide (sphingolipid) acquisition

To measure the intra-inclusion accumulation of ceramide (and/or ceramide-derived lipids), cells were seeded in 96-well plates and infected (for the experiment shown in Fig 5D, mCherry-expressing derivatives of the indicated strains were used). At 14 hpi, cells were incubated for 15 min at 4°C, rinsed with cold HBSS (Gibco), and then incubated for 30 min at 4°C in HBSS containing 5 µM NBD C6-ceramide complexed to BSA (ThermoFisher). Subsequently, the cells were rinsed twice with growth medium and incubated for another 6 hours in medium (37°C, 5% CO_2). BFA (3 µg/ml) was added to some wells at 12 hpi and was also present during the incubation with ceramide, but not thereafter. After the 6-hour incubation period, cells were stained with Hoechst 33342 (2 µg/ml, 10 min), washed twice with growth medium, and imaged live with an automated fluorescence microscope (ImageXpress Micro XL, Molecular Devices) at about 21 hpi. Inclusions were detected in MetaXpress (Molecular Devices) based on mCherry fluorescence (in experiments using mCherry-expressing strains) or NBD fluorescence (in all other experiments). The average intra-inclusion NBD fluorescence intensity was determined and

normalized to the average intra-inclusion NBD fluorescence observed during infection with CTL2.

siRNA-mediated gene silencing

Cells were transfected with siRNAs (Dharmacon siGENOME Human SMART pools, *i.e.* a mix of four gene-specific siRNAs, Table S4) at a concentration of 25 nM using DharmaFECT-1 transfection reagent (Dharmacon). To reduce toxicity, the transfection medium containing siRNAs and reagent was removed after a 6-hour incubation and replaced with fresh growth medium. In control transfections, RNA-free siRNA buffer (Dharmacon) was added instead of siRNAs. To determine knockdown efficiencies by western blotting, protein samples were prepared (as described below) at 46 hours post transfection. To determine effects of knockdowns on the IFN response during infection, cells were infected at 32 hours (reporter cell assay) or at 36 hours (detection of STING/IRF3 phosphorylation) post transfection with siRNAs and then further processed at 14 hpi. To determine effects of knockdowns on the IFN response after DNA stimulation (detection of STING/IRF3 phosphorylation), cells were transfected with DNA at 46 hours post transfection with siRNAs and then further processed four hours later. To test the effect of Rab35 depletion on Rab35 effector recruitment to inclusions, cells were transfected with Rab35 effector expression plasmids at 48 hours post transfection with siRNAs, infected five hours later, and analyzed at 24 hpi.

Fluorescence microscopy

To preserve cells for fluorescence microscopy, cells were fixed by a 10-min incubation in cold (-20°C) methanol or a 20-min incubation in 2-4% formaldehyde (with or without 0.025% glutaraldehyde) at room temperature. Formaldehyde-fixed cells (but not methanol-fixed cells) were then permeabilized for 15 min with 0.2% Triton X-100 in Dulbecco's phosphate-buffered saline (DPBS; Gibco). Subsequently, the cells were incubated for 20 min in blocking solution (2-3% BSA in DPBS), and then for 1 hour with blocking solution containing primary antibodies (Table S5). Cells were then washed thrice with DPBS, incubated for 1 hour in blocking solution containing Hoechst 33258/33342 (Invitrogen; 2-10 µg/ml) and AlexaFluor (488, 555, or 647) -labeled secondary antibodies (Invitrogen; 1:250-1:1000), and then washed again thrice with DPBS. Cells grown and stained on glass coverslips were transferred to microscope slides and embedded in Mowiol (18 ml 0.13 M Tris/HCl (pH 8.5), 2.4 g polyvinyl alcohol, 6 g glycerin, 0.01% p-phenylenediamine-dihydrochloride) (3) or ProLong Glass Antifade Mountant (Invitrogen). Images were taken with various microscopy systems, including epifluorescence microscopes (Zeiss Axio Observer.Z1, Zeiss Axio Imager.Z2), confocal microscopes (Zeiss LSM 780, Leica

SP8), and high-content imaging platforms (ImageXpress Micro XL system (Molecular Devices), Cellomics ArrayScan VTI HCS (ThermoFisher)). The percentage of cells displaying STING translocation (accumulation of STING-HA in post-ER vesicles; as previously shown (3)) recruitment of EGFP-Rab fusion proteins to the inclusion (enrichment at inclusion membrane; examples shown in Fig 5A), or recruitment of HA-tagged Rab effector proteins to the inclusion, was determined by manual inspection of images taken with the ImageXpress Micro XL or Zeiss Axio Imager.Z2 systems. Except for the assessment of Rab effector recruitment, this analysis was conducted blinded, *i.e.* the individual conducting the counting was unaware of the sample identity at the time of counting.

Co-immunoprecipitation

HeLa cells were infected with the indicated *Chlamydia* strains and when indicated transfected with EGFP-Rab expression plasmids at 1 hpi (see above). At 26 hpi, cells were lysed in Pierce IP lysis buffer containing Pierce Protease and Phosphatase Inhibitor (ThermoFisher). Lysates were homogenized by passing through a needle (0.33 x 12 mm) and cleared by centrifugation (17000-20000 x g, 4°C, 20 min). For immunoprecipitation of FLAG-tagged proteins, ANTI-FLAG M2 magnetic beads (Merck) were incubated with the lysate (500-800 µl, 0.6-1.0 mg/ml protein) for 6 hours at 4°C and then washed four times with Pierce IP lysis buffer. Proteins were eluted by incubation in 0.1 M glycine/HCl (pH 3.0) followed by neutralization by addition of 1/5 volume of 0.5 M Tris/HCl, 1.5 M NaCl (pH 7.4). For the co-immunoprecipitation of IPAM, proteins were instead eluted by boiling (3 min, 100°C) of the beads in Laemmli buffer (final concentration: 50 mM Tris (pH 6.8), 10% glycerol, 2% SDS, 50 mM DTT, traces of bromophenol blue). For immunoprecipitation of EGFP-fusion proteins, GFP-Trap_MA magnetic beads (Chromotek) were incubated with lysate (1350 µl, 0.6 mg/ml protein; diluted in TBS/EDTA buffer (10 mM Tris/HCl, 150 mM NaCl, 0.5 mM EDTA pH 7.5)) for 5 hours at 4°C and then washed four times with TBS/EDTA buffer. Proteins were eluted by boiling, as described above. Aliquots from the initial lysate (lysate fraction, also known as input), lysate after incubation with beads (unbound fraction), and the buffer used for the last wash step (wash fraction) were also collected. Samples were stored at -20°C until analysis by western blotting.

Western blot analysis

Protein extracts were prepared by cell lysis in boiling 1% SDS buffer (50 mM Tris/HCl (pH 7.5), 1% SDS, 0.1 M NaCl), as previously described (3), quantified using a BCA protein assay (Pierce), and adjusted to equal protein content by dilution with 1% SDS buffer. Protein extracts (and Co-IP samples) were then mixed with loading buffer (Laemmli buffer (see above) or

NuPAGE LDS sample buffer containing NuPAGE reducing agent (ThermoFisher)). Subsequently, the samples were denatured (10 min, 95-100°C), before separation by SDS PAGE using commercial gels (NuPAGE Novex 4-12% Bis-Tris gels (ThermoFisher) or Mini-PROTEAN TGX 4-20% gels (Bio-Rad)) and transfer to 0.2 µm nitrocellulose membranes (Bio-Rad). Membranes were blocked (5% milk or 3% BSA in TBST buffer (20 mM Tris (pH 7.5), 150 mM NaCl, 0.1% Tween 20)) for 1 hour, incubated o/n at 4°C with blocking solution containing primary antibodies (Table S5), washed thrice with TBST, incubated for 1 hour with HRP-conjugated secondary antibodies (Southern Biotech or ThermoFisher, 1:10000-1:50000 diluted in blocking solution), and washed again thrice with TBST. Membranes were then incubated for 1 min with HRP substrate (ECL Prime (GE Healthcare) or SuperSignal West Pico PLUS (ThermoFisher)) and chemiluminescent signals were recorded with an Image Quant LAS4000 imaging system (GE Healthcare) or an Amersham Imager 680RGB (GE Healthcare). Membranes were stripped with Restore Plus western blot stripping buffer (ThermoFisher) and blocked before detection of additional targets. Band intensities were quantified using Image Quant TL (GE Healthcare). Expression levels of bacterial and host proteins were normalized to the expression of the *Chlamydia* protein Slc1 and host β-actin, respectively. Levels of phosphorylated Src were instead normalized to total Src.

Statistical analysis

Statistical analysis was conducted in GraphPad Prism 8. The following statistical tests were used when indicated in the figure legends: one-way ANOVA (with Newman-Keuls post hoc test) and student t test (unpaired two-tailed, assuming equal variance). The following significance levels were considered: *, $p<0.05$; **, $p<0.01$; *** $p<0.001$; ns, not significant.

References

1. Barker JR, Koestler BJ, Carpenter VK, Burdette DL, Waters CM, Vance RE, Valdivia RH. 2013. STING-dependent recognition of cyclic di-AMP mediates type I interferon responses during *Chlamydia trachomatis* infection. mBio 4:e00018-13.
2. Buckner LR, Schust DJ, Ding J, Nagamatsu T, Beatty W, Chang TL, Greene SJ, Lewis ME, Ruiz B, Holman SL, Spagnuolo RA, Pyles RB, Quayle AJ. 2011. Innate immune mediator profiles and their regulation in a novel polarized immortalized epithelial cell model derived from human endocervix. J Reprod Immunol 92:8-20.
3. Sixt BS, Bastidas RJ, Finethy R, Baxter RM, Carpenter VK, Kroemer G, Coers J, Valdivia RH. 2017. The *Chlamydia trachomatis* inclusion membrane protein CpoS counteracts STING-mediated cellular surveillance and suicide programs. Cell Host Microbe 21:113-121.

4. van Kuppeveld FJ, van der Logt JT, Angulo AF, van Zoest MJ, Quint WG, Niesters HG, Galama JM, Melchers WJ. 1992. Genus- and species-specific identification of mycoplasmas by 16S rRNA amplification. Appl Environ Microbiol 58:2606-15.
5. Rzomp KA, Scholtes LD, Briggs BJ, Whittaker GR, Scidmore MA. 2003. Rab GTPases are recruited to chlamydial inclusions in both a species-dependent and species-independent manner. Infect Immun 71:5855-70.
6. Huang B, Hubber A, McDonough JA, Roy CR, Scidmore MA, Carlyon JA. 2010. The *Anaplasma phagocytophilum*-occupied vacuole selectively recruits Rab-GTPases that are predominantly associated with recycling endosomes. Cell Microbiol 12:1292-307.
7. Sayers EW, Bolton EE, Brister JR, Canese K, Chan J, Comeau DC, Connor R, Funk K, Kelly C, Kim S, Madej T, Marchler-Bauer A, Lanczycki C, Lathrop S, Lu Z, Thibaud-Nissen F, Murphy T, Phan L, Skripchenko Y, Tse T, Wang J, Williams R, Trawick BW, Pruitt KD, Sherry ST. 2022. Database resources of the national center for biotechnology information. Nucleic Acids Res 50:D20-D26.
8. Wang X, Hybiske K, Stephens RS. 2018. Direct visualization of the expression and localization of chlamydial effector proteins within infected host cells. Pathog Dis 76:fty011.
9. Agaisse H, Derre I. 2013. A *C. trachomatis* cloning vector and the generation of *C. trachomatis* strains expressing fluorescent proteins under the control of a *C. trachomatis* promoter. PLoS One 8:e57090.
10. Johnson CM, Fisher DJ. 2013. Site-specific, insertional inactivation of incA in *Chlamydia trachomatis* using a group II intron. PLoS One 8:e83989.
11. Filcek K, Vielfort K, Muraleedharan S, Henriksson J, Valdivia RH, Bavoil PM, Sixt BS. 2019. Insertional mutagenesis in the zoonotic pathogen *Chlamydia caviae*. PLoS One 14:e0224324.
12. Wang Y, Kahane S, Cutcliffe LT, Skilton RJ, Lambden PR, Clarke IN. 2011. Development of a transformation system for *Chlamydia trachomatis*: restoration of glycogen biosynthesis by acquisition of a plasmid shuttle vector. PLoS Pathog 7:e1002258.
