# Supplementary figures and images for: The Chlamydia effector CpoS modulates the inclusion microenvironment and restricts the interferon response by acting on Rab35
Source: mBio. 2023 Aug 2;14(4):e03190-22. doi: 10.1128/mbio.03190-22 (PMC10470785; doi:10.1128/mbio.03190-22)

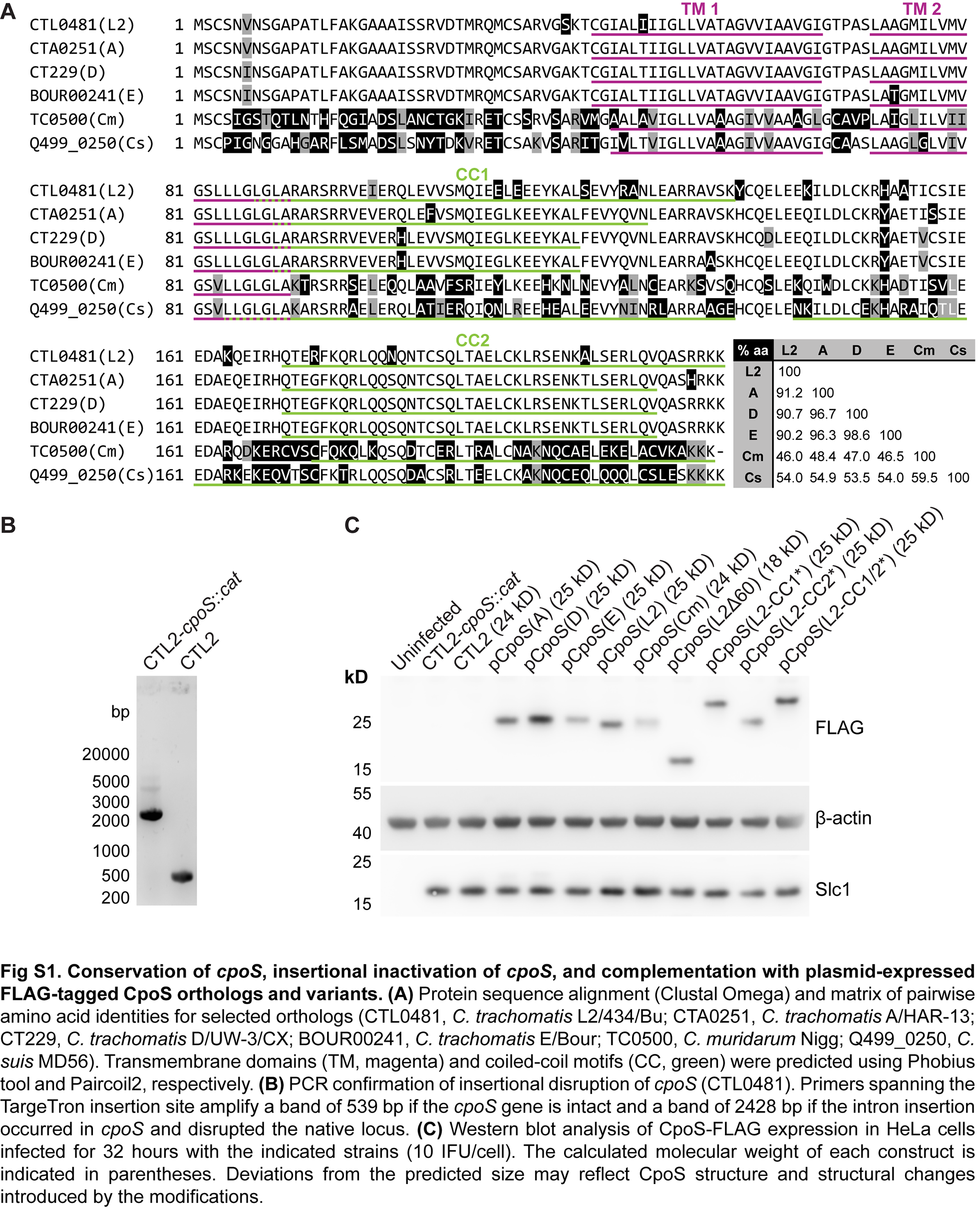

Supplement: Figure S1 — Conservation of cpoS, insertional inactivation of cpoS, and complementation with plasmid-expressed FLAG-tagged CpoS orthologs and variants. [file mbio.03190-22-s0002.tif]

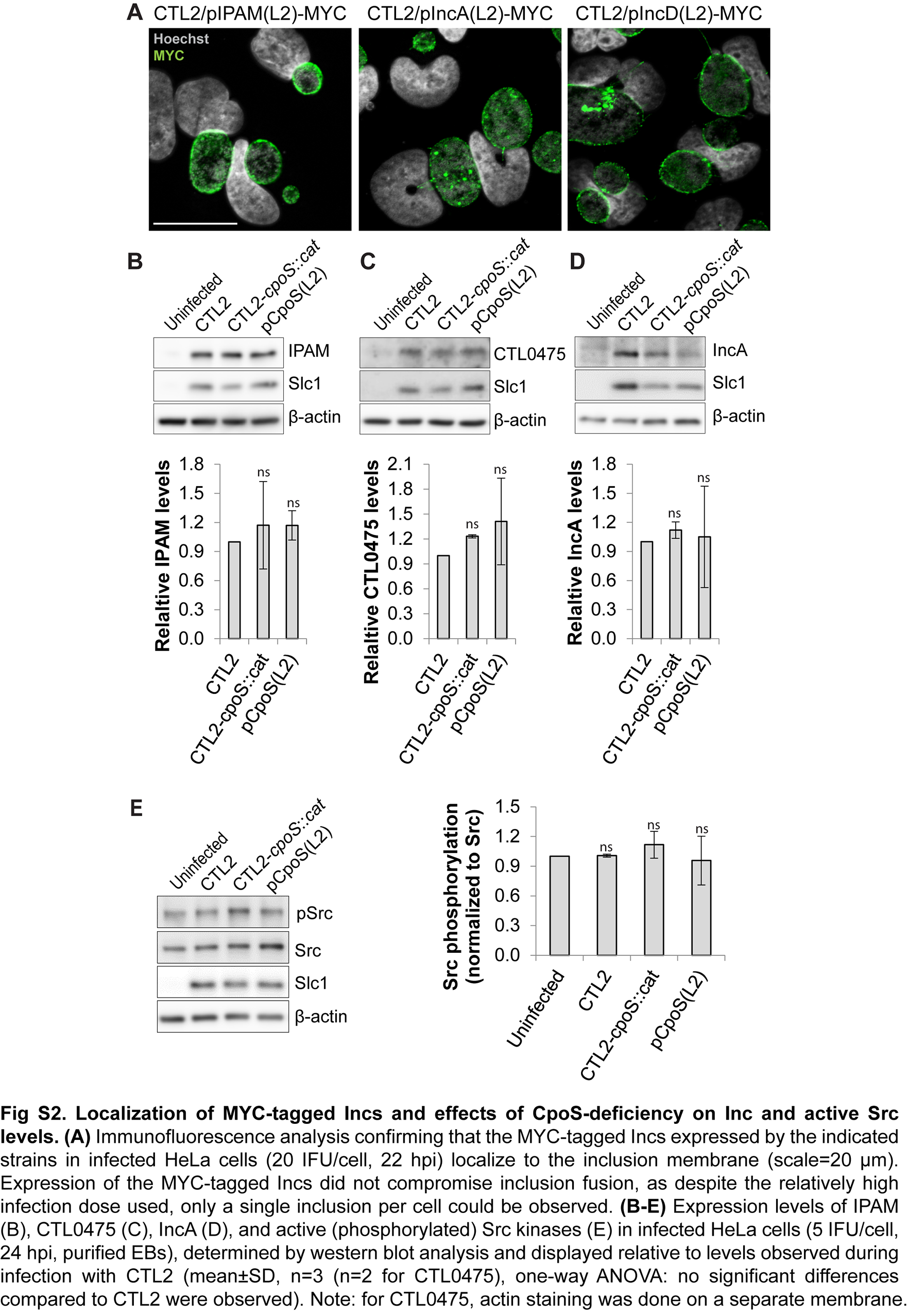

Supplement: Figure S2 — Localization of MYC-tagged Incs and effects of CpoS-deficiency on Inc and active Src levels. [file mbio.03190-22-s0003.tif]

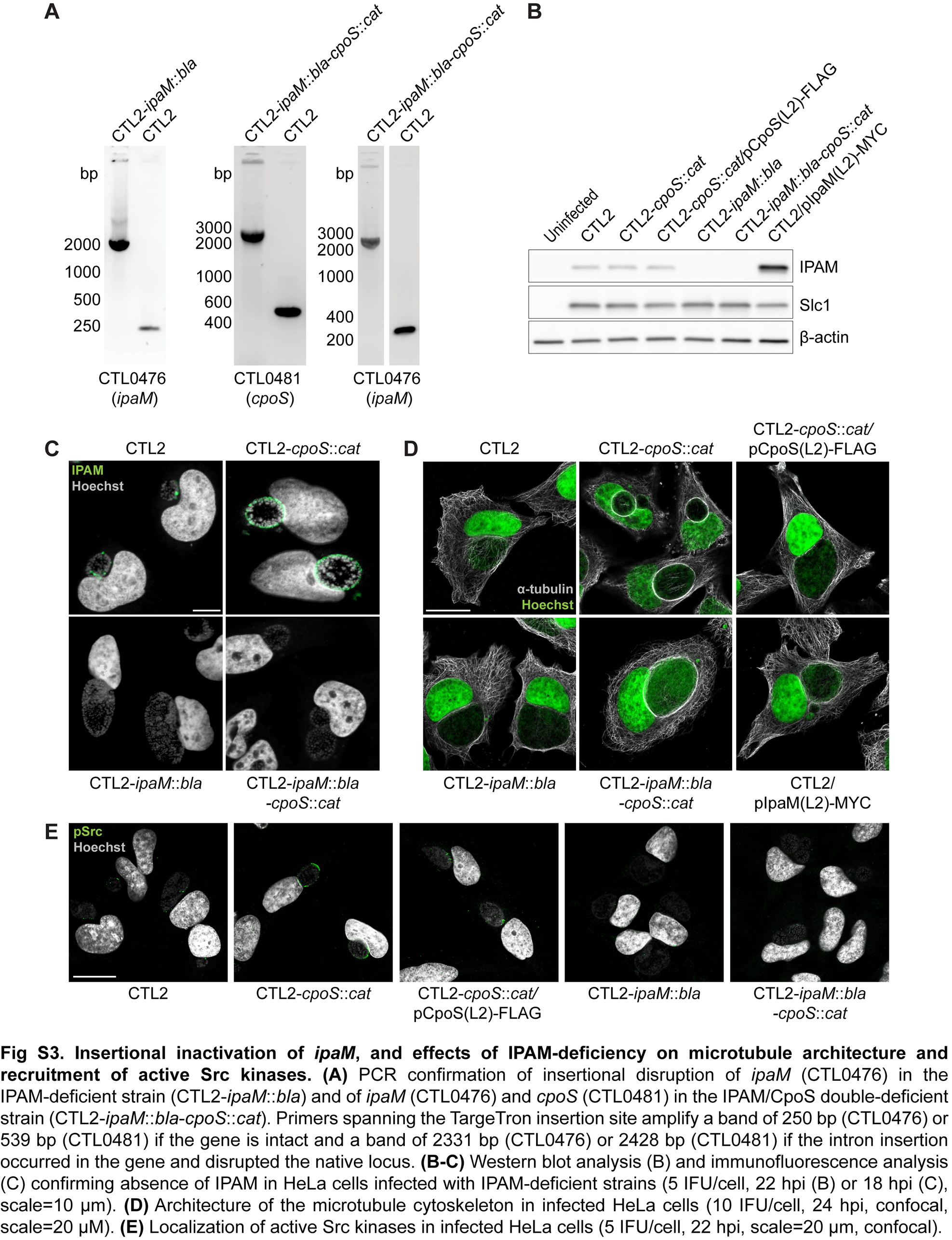

Supplement: Figure S3 — Insertional inactivation of ipaM, and effects of IPAM-deficiency on microtubule architecture and recruitment of active Src kinases. [file mbio.03190-22-s0004.tif]

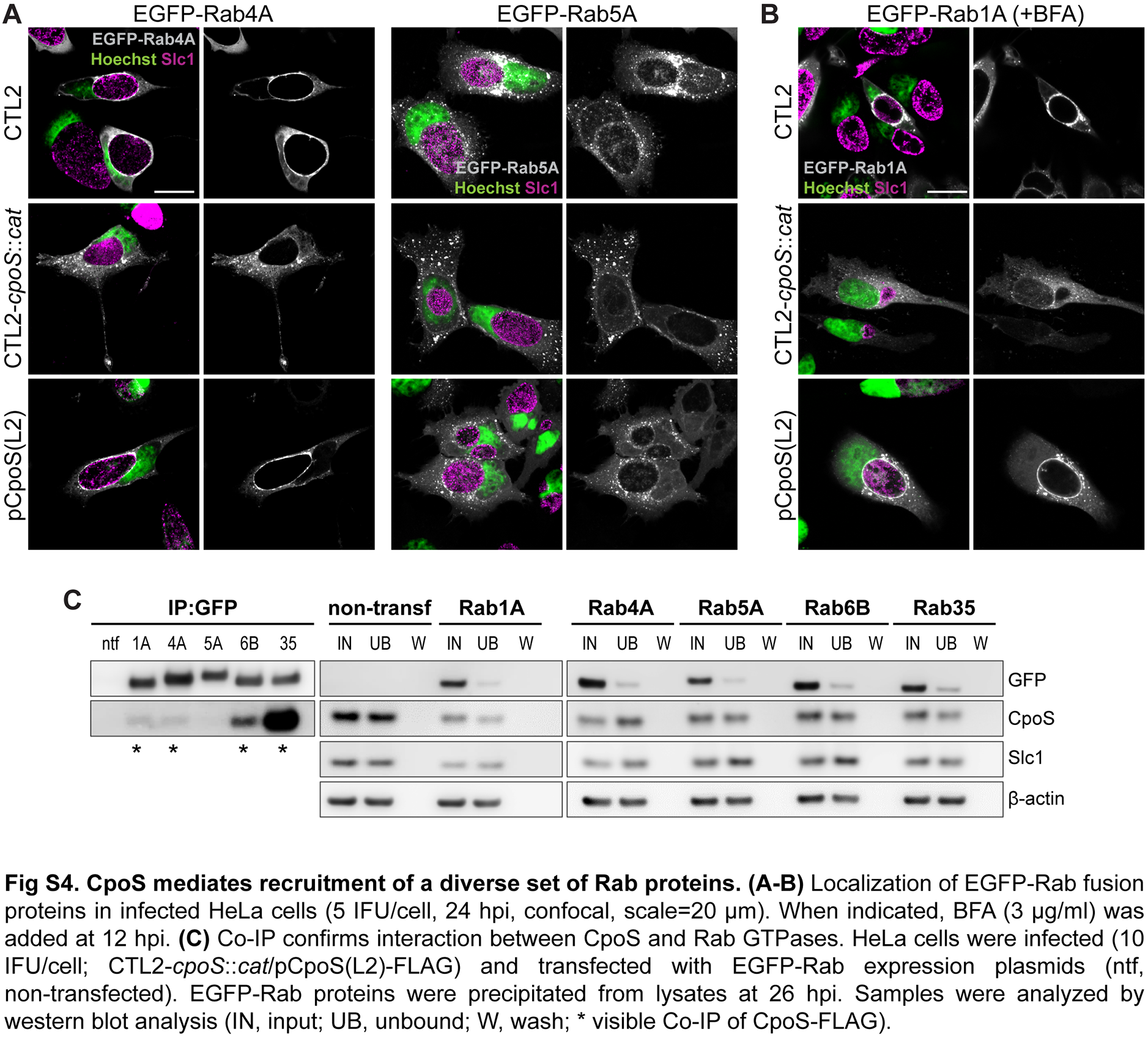

Supplement: Figure S4 — CpoS mediates recruitment of a diverse set of Rab proteins. [file mbio.03190-22-s0005.tif]

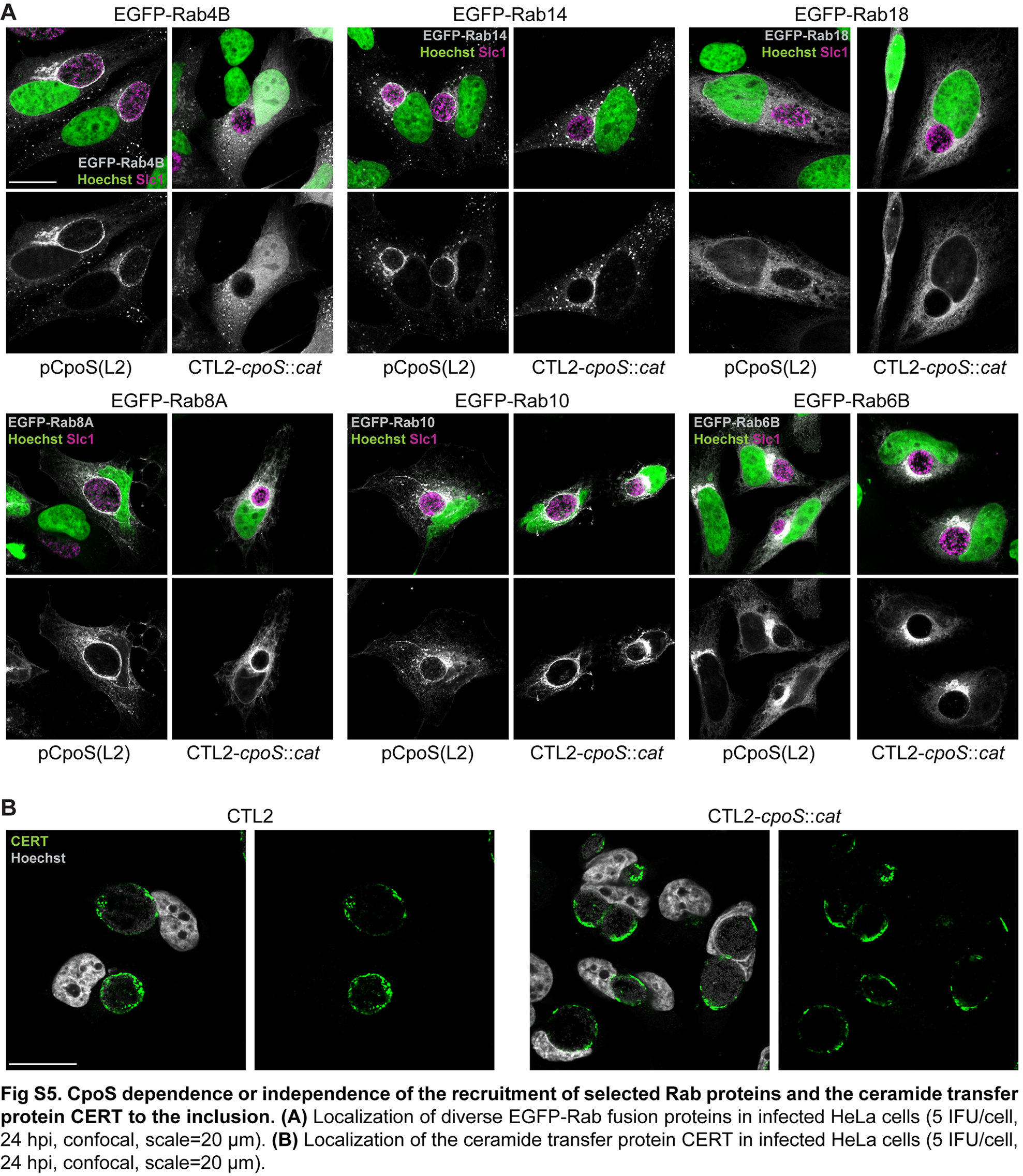

Supplement: Figure S5 — CpoS dependence or independence of the recruitment of selected Rab proteins and the ceramide transfer protein CERT to the inclusion. [file mbio.03190-22-s0006.tif]

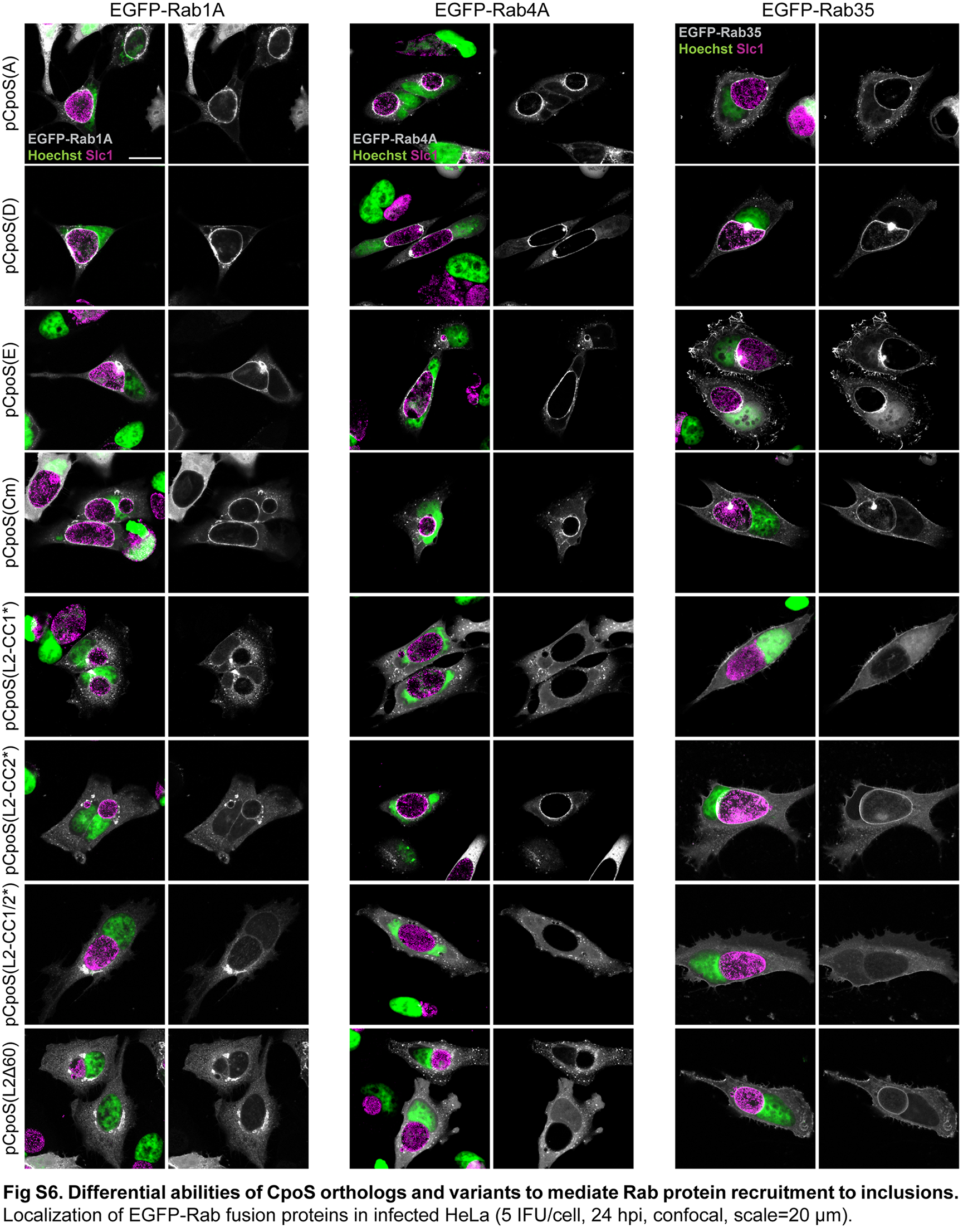

Supplement: Figure S6 — Differential abilities of CpoS orthologs and variants to mediate Rab protein recruitment to inclusions. [file mbio.03190-22-s0007.tif]

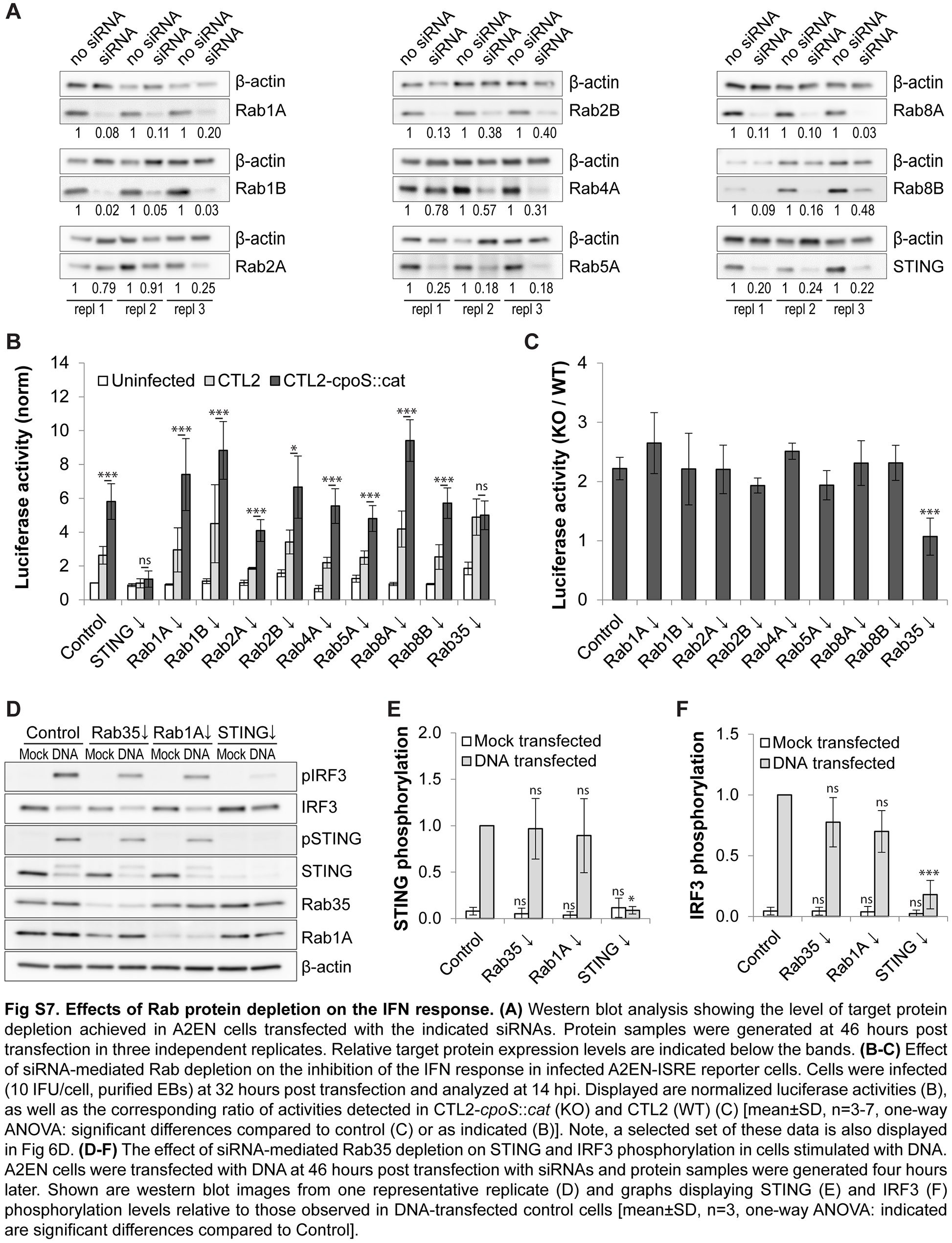

Supplement: Figure S7 — Effects of Rab protein depletion on the IFN response. [file mbio.03190-22-s0008.tif]

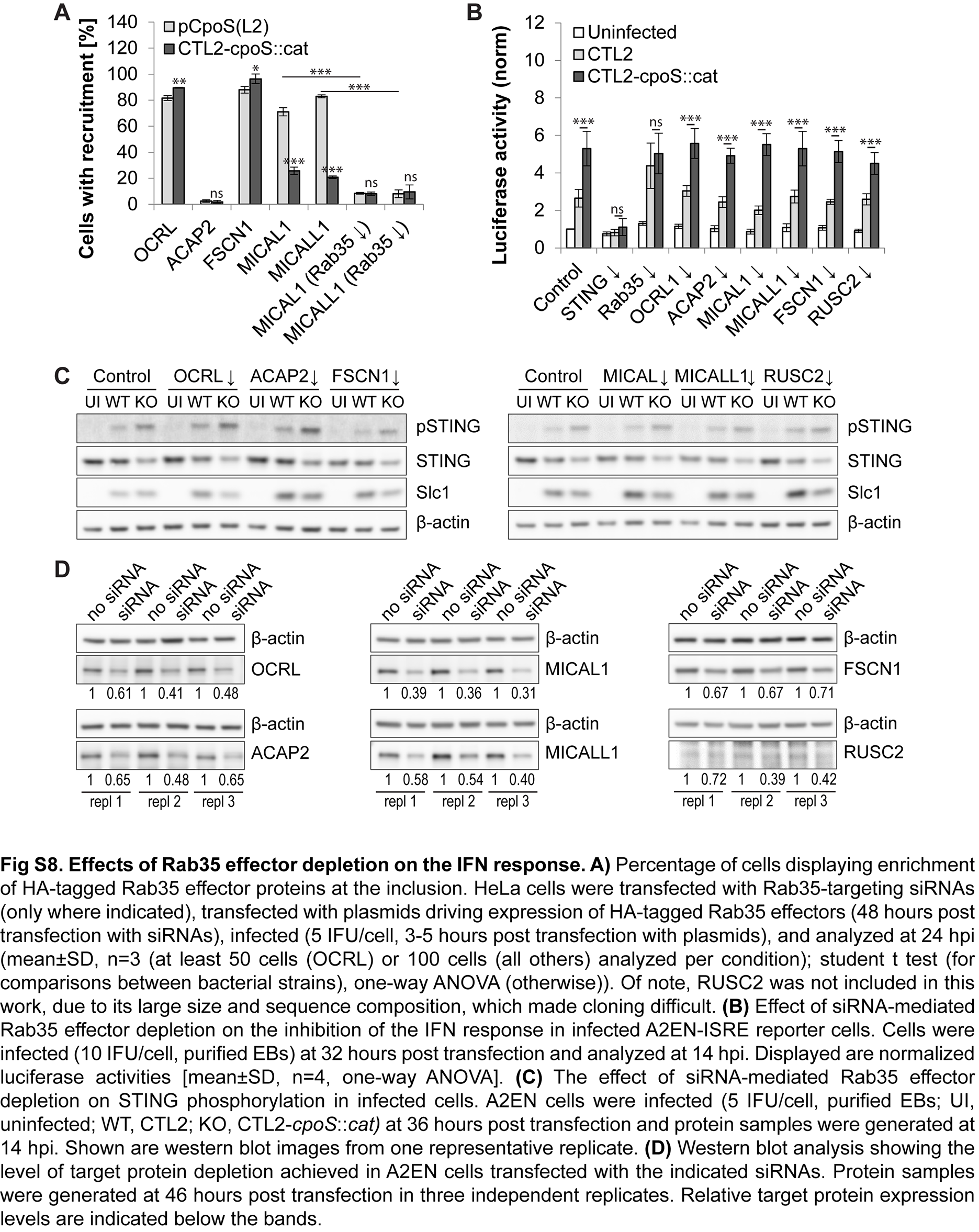

Supplement: Figure S8 — Effects of Rab35 effector depletion on the IFN response. [file mbio.03190-22-s0009.tif]
