# Supplementary material for: The Chlamydia effector CpoS modulates the inclusion microenvironment and restricts the interferon response by acting on Rab35
Source: mBio. 2023 Aug 2;14(4):e03190-22. doi: 10.1128/mbio.03190-22 (PMC10470785; doi:10.1128/mbio.03190-22)
Supplement: Supplemental Tables — Tables S1-S5. [file mbio.03190-22-s0010.pdf]

Table S1. Designations of Inc proteins mentioned in this study. Listed are CTL and CT locus tag designations (referring to genes in *C. trachomatis* L2/434/Bu and *C. trachomatis* D/UW-3/Cx, respectively) of all Incs mentioned. For CpoS, designations of additional orthologs mentioned in the study are listed as well.

**Inc protein**	***Chlamydia* strain**	**Locus tag**	**Other designation used in this study for simplicity**
CpoS	*C. trachomatis* L2/434/Bu	CTL0481	CpoS(L2)
	C. trachomatis D/UW-3/Cx	CT229	CpoS(D)
	C. trachomatis A/HAR-13	CTA0251	CpoS(A)
	C. trachomatis E/Bour	BOUR00241	CpoS(E)
	C. muridarum Nigg	TC0500	CpoS(Cm)
	C. suis MD56	Q499_0250	CpoS(Cs)
IPAM	*C. trachomatis* L2/434/Bu	CTL0476	IPAM(L2)
	C. trachomatis D/UW-3/Cx	CT223	IPAM(D)
IncA	*C. trachomatis* L2/434/Bu	CTL0374	IncA(L2)
	C. trachomatis D/UW-3/Cx	CT119	IncA(D) – *term not used explicitly in this study*
IncB	*C. trachomatis* L2/434/Bu	CTL0484	IncB(L2) – *term not used explicitly in this study*
	C. trachomatis D/UW-3/Cx	CT232	IncB(D) – *term not used explicitly in this study*
IncD	*C. trachomatis* L2/434/Bu	CTL0370	IncD(L2)
	C. trachomatis D/UW-3/Cx	CT115	IncD(D)
unnamed	*C. trachomatis* L2/434/Bu	CTL0475	
	C. trachomatis D/UW-3/Cx	CT222	

Table S2. *Chlamydia* strains used in this study.

Strain name	Short name	Description	CpoS variant expressed
CTL2	CTL2	*C. trachomatis* strain L2/434/Bu	Endogenous CpoS(L2)
CTL2-*cpoS*::*cat*	CTL2-*cpoS*::*cat*	Derived from CTL2, *cat* insertion in *cpoS*	CpoS(L2) truncated after aa 92
CTL2-*ipaM*::*bla*	CTL2-*ipaM*::*bla*	Derived from CTL2, *bla* insertion in *ipaM*	Endogenous CpoS(L2)
CTL2-*ipaM*::*bla*-*cpoS*::*cat*	CTL2-*ipaM*::*bla*-*cpoS*::*cat*	Derived from CTL2, *bla* insertion in *ipaM, cat* insertion in *cpoS*	CpoS(L2) truncated after aa 92
CTL2-*cpoS*::*cat* / pCpoS(L2)-FLAG	pCpoS(L2)	Derived from CTL2-*cpoS*::*cat*, transformed with plasmid enabling expression of FLAG-tagged CpoS from *C. trachomatis* strain L2/434/Bu (CTL0481)	Plasmid-expressed CpoS(L2)-FLAG
CTL2-*cpoS*::*cat* / pCpoS(A)-FLAG	pCpoS(A)	Derived from CTL2-*cpoS*::*cat*, transformed with plasmid enabling expression of FLAG-tagged CpoS from *C. trachomatis* strain A/HAR-13 (CTA0251)	Plasmid-expressed CpoS(A)-FLAG
CTL2-*cpoS*::*cat* / pCpoS(D)-FLAG	pCpoS(D)	Derived from CTL2-*cpoS*::*cat*, transformed with plasmid enabling expression of FLAG-tagged CpoS from *C. trachomatis* strain D/UW-3/CX (CT229)	Plasmid-expressed CpoS(D)-FLAG
CTL2-*cpoS*::*cat* / pCpoS(E)-FLAG	pCpoS(E)	Derived from CTL2-*cpoS*::*cat*, transformed with plasmid enabling expression of FLAG-tagged CpoS from *C. trachomatis* strain E/Bour (BOUR00241)	Plasmid-expressed CpoS(E)-FLAG
CTL2-*cpoS*::*cat* / pCpoS(Cm)-FLAG	pCpoS(Cm)	Derived from CTL2-*cpoS*::*cat*, transformed with plasmid enabling expression of FLAG-tagged CpoS from *C. muridarum* strain Nigg (TC0500)	Plasmid-expressed CpoS(Cm)-FLAG
CTL2-*cpoS*::*cat* / pCpoS(L2Δ60)-FLAG	pCpoS(L2Δ60)	Derived from CTL2-*cpoS*::*cat*, transformed with plasmid enabling expression of FLAG-tagged CpoS from *C. trachomatis* strain L2/434/Bu (CTL0481) with a 60 aa C-terminal deletion	Plasmid-expressed CpoS(L2Δ60)-FLAG
CTL2-*cpoS*::*cat* / pCpoS(L2-CC1*)-FLAG	pCpoS(L2-CC1*)	Derived from CTL2-*cpoS*::*cat*, transformed with plasmid enabling expression of FLAG-tagged CpoS from *C. trachomatis* strain L2/434/Bu (CTL0481) with two point mutations in CC1 (I99P + L113P)	Plasmid-expressed CpoS(L2-CC1*)-FLAG (=CpoS(L2-I99P-L113P)-FLAG)
CTL2-*cpoS*::*cat* / pCpoS(L2-CC2*)-FLAG	pCpoS(L2-CC2*)	Derived from CTL2-*cpoS*::*cat*, transformed with plasmid enabling expression of FLAG-tagged CpoS from *C. trachomatis* strain L2/434/Bu (CTL0481) with two point mutations in CC2 (N181P + L188P)	Plasmid-expressed CpoS(L2-CC2*)-FLAG (=CpoS(L2-N181P-L188P)-FLAG)
CTL2-*cpoS*::*cat* / pCpoS(L2-CC1/2*)-FLAG	pCpoS(L2-CC1/2*)	Derived from CTL2-*cpoS*::*cat*, transformed with plasmid enabling expression of FLAG-tagged CpoS from *C. trachomatis* strain L2/434/Bu (CTL0481) with two point mutations in CC1 (I99P + L113P) and two point mutations in CC2 (N181P + L188P)	Plasmid-expressed CpoS(L2-CC1/2*)-FLAG (=CpoS(L2-I99P-L113P-N181P-L188P)-FLAG)

Table S2 (continued). ***Chlamydia*** **strains used in this study.**

Strain name	Short name	Description	CpoS variant expressed
CTL2 / pIPAM(L2)-MYC	pIPAM(L2)-MYC	Derived from CTL2, transformed with plasmid enabling expression of MYC-tagged IPAM from *C. trachomatis* strain L2/434/Bu (CTL0476)	Endogenous CpoS(L2)
CTL2 / pIncA(L2)-MYC	pIncA(L2)-MYC	Derived from CTL2, transformed with plasmid enabling expression of MYC-tagged IncA from *C. trachomatis* strain L2/434/Bu (CTL0374)	Endogenous CpoS(L2)
CTL2 / pIncD(L2)-MYC	pIncD(L2)-MYC	Derived from CTL2, transformed with plasmid enabling expression of MYC-tagged IncD from *C. trachomatis* strain L2/434/Bu (CTL0370)	Endogenous CpoS(L2)
CTL2 / p2TK2-SW2-mCherry	n/a	Derived from CTL2, transformed with plasmid enabling expression of mCherry	Endogenous CpoS(L2)
CTL2-*cpoS*::*cat* / p2TK2-SW2-mCherry	n/a	Derived from CTL2-*cpoS*::*cat*, transformed with plasmid enabling expression of mCherry	CpoS(L2) truncated after aa 92
CTL2 / pTL2-tetO-IncB-GFP11x7-FLAG	CTL2/pIncB-GFP11	Derived from CTL2, transformed with plasmid enabling expression of IncB tagged with seven repeats of GFP11 and a FLAG tag	Endogenous CpoS(L2)
CTL2-*cpoS*::*cat* / pTL2-tetO-IncB-GFP11x7- FLAG	CTL2-*cpoS*::*cat*/pIncB-GFP11	Derived from CTL2-*cpoS*::*cat*, transformed with plasmid enabling expression of IncB tagged with seven repeats of GFP11 and a FLAG tag	CpoS(L2) truncated after aa 92

Table S3. Primers used in this study.

Purpose	Primer name	Primer sequence (5'→ 3')
Confirmation of cloned EGFP-Rab constructs	pEGFPC1for	GATCACTCTCGGCATGGAC
Retargeting of vector pDFTT3 for disruption of CTL0476	IBS-CTL0476	AAAAAAGCTTATAATTATCCTTACTTCTCTTCTTTGTGCGCCCAGATAGGGTG
	EBS1d-CTL0476	CAGATTGTACAAATGTGGTGATAACAGATAAGTCTTCTTTTATAACTTACCTTTCTTTGT
	EBS2-CTL0476	TGAACGCAAGTTTCTAATTTCGGTTAGAAGTCGATAGAGGAAAGTGTCT
Verification of intron insertion into CTL0476	CTL0476_seqF	GACTTGCACACTTAGTATCC
	CTL0476_seqR	GGTGGTGCTGTTGTATTGG
Verification of intron insertion into CTL0481	CTL0481_seqF	CTCTGCTGTTAATTGACTGC
	CTL0481_seqR	GCTACTCTTTTCGCAAAAGG
Amplification of CpoS orthologs and variants for insertion into p2TK2-SW2	CTL0481_F [a]	GGTACCGGTCTGAGAGGGGGTATATCTTTTG
	CTL0481_R [b]	GCAGGTACCTTTTTTACGACGGGATGCCTG
	CTL0481Δ60_R [c]	GCAGGTACCTGTTGCGG CATGACGCTTAC
	CTA0251_R [d]	GCAGGTACCTTTTTTACGATGGGATGCCTG
	TC0500_F [e]	GGTACCGGTTAGAGAGTCTTTTGGGACTAAATG
	TC0500_R [f]	GCAGGTACCCTTCTTCTTAGCTTTAACACA
Amplification of IPAM-MYC for insertion into p2TK2-SW2	CTL0476-MYC_F	CTCGTCCACGGTCACCGGTGTTCGATTTTAAGATTCTTAGTGCAATG
	CTL0476-MYC_R	GCAGCTAGCTTACAGATCCTCTTCTGAGATGAGTTTTTGTTCCACCCGAGAGCCGTAATTGAAATC
Amplification of IncA-MYC for insertion into p2TK2-SW2	CTL0374-MYC_F	CTAGGTGCCACGGTACCGGTATCGTTAATCCAGTCAACAATATTAAT
	CTL0374-MYC_R	TTAGCTAGCTTACAGATCCTCTTCTGAGATGAGTTTTTGTTCGGAGCTTTTTGTAGAGGGTGATGC
Amplification of IncD-MYC for insertion into p2TK2-SW2	CTL0370-MYC_F	CTAAGTTCCACGGTACCGGTGCTATCTCTTGTTGCTCCGAGTTAAAC
	CTL0370-MYC_R	TTAGCTAGCTTACAGATCCTCTTCTGAGATGAGTTTTTGTTCGCTCGCCCCTTTTTTACTCACCGA
Amplification of OCRL-HA for insertion into pcDNA3.1	OCRL_F	ATAGGATCCATGGAGCCGCCGCTCCCGGT
	OCRL_R	ATAGCGGCCGCTTAAGCGTAATCTGGAACATCGTATGGGTAGTCTTCTTCGCTCCCAAGCA
Amplification of ACAP2-HA for insertion into pcDNA3.1	ACAP2_F	GCCGCGGATCCATGAAGATGACTGTGGATTTCG
	ACAP2_R	ATAGCGGCCGCTTAAGCGTAATCTGGAACATCGTATGGGTAGAATTTCTGTGAATCTTGCT

[a] Forward primer used for amplification of CTL0481 and CTL0481Δ60 (*C. trachomatis* L2/434/Bu), CTA0251 (*C. trachomatis* A/HAR-13), CT229 (*C. trachomatis* D/UW-3/CX), and BOUR00241 (*C. trachomatis* E/Bour). [b] Reverse primer used for amplification of CTL0481(*C. trachomatis* L2/434/Bu), CT229 (*C. trachomatis* D/UW-3/CX), and BOUR00241 (*C. trachomatis* E/Bour). [c] Reverse primer used for amplification of CTL0481Δ60 (*C. trachomatis* L2/434/Bu). [d] Reverse primer used for amplification of CTA0251 (*C. trachomatis* A/HAR-13). [e] Forward primer used for amplification of TC0500 (*C. muridarum* Nigg). [f] Reverse primer used for amplification of TC0500 (*C. muridarum* Nigg).

Table S4. siRNAs used in this study.

Reagent	Vendor	Catalogue Number
siGENOME siRNA SMARTpool targeting human STING	Dharmacon	M-024333-00-0005
siGENOME siRNA SMARTpool targeting human Rab1A	Dharmacon	M-008283-01-0005
siGENOME siRNA SMARTpool targeting human Rab1B	Dharmacon	M-008958-01-0005
siGENOME siRNA SMARTpool targeting human Rab2A	Dharmacon	M-010533-01-0005
siGENOME siRNA SMARTpool targeting human Rab2B	Dharmacon	M-010370-00-0005
siGENOME siRNA SMARTpool targeting human Rab4A	Dharmacon	M-008539-01-0005
siGENOME siRNA SMARTpool targeting human Rab5A	Dharmacon	M-004009-00-0005
siGENOME siRNA SMARTpool targeting human Rab8A	Dharmacon	M-003905-00-0005
siGENOME siRNA SMARTpool targeting human Rab8B	Dharmacon	M-008744-01-0005
siGENOME siRNA SMARTpool targeting human Rab35	Dharmacon	M-009781-00-0005
siGENOME siRNA SMARTpool targeting human OCRL	Dharmacon	M-010026-01-0005
siGENOME siRNA SMARTpool targeting human ACAP2	Dharmacon	M-012811-00-0005
siGENOME siRNA SMARTpool targeting human MICAL1	Dharmacon	M-010192-00-0005
siGENOME siRNA SMARTpool targeting human MICALL1	Dharmacon	M-015102-01-0005
siGENOME siRNA SMARTpool targeting human FSCN1	Dharmacon	M-019576-01-0005
siGENOME siRNA SMARTpool targeting human RUSC2	Dharmacon	M-026133-01-0005

Table S5. Primary antibodies used in this study.

Reagent	Source	Dilution used in microscopy	Dilution used in western blot
mouse-anti-FLAG	Merck (F1804)	1:250-1:500	
mouse-anti-FLAG	Merck (F3165)	1:250-1:500	1:1000
rabbit-anti-FLAG	Merck (F7425)		1:1000-1:5000
rabbit-anti-FLAG	Cell signaling (14793)		1:1000
rabbit-anti-MYC	Cell Signaling (2278)	1:500	1:1000
rat-anti-HA	Merck (11867423001)	1:500	
rabbit-anti-GFP	Cell Signaling (2956)		1:1000
mouse-anti-β-actin	Cell Signaling (3700)		1:2000-1:5000
mouse-anti-β-actin (HRP-labeled)	Abcam (ab49900)		1:50,000
mouse-anti-α-tubulin	Merck (T5168)	1:250-1:1000	
rabbit-anti-Src	Cell Signaling (2123)		1:1000
rabbit-anti-phospho(Tyr416)-Src	Cell Signaling (6943)		1:1000
mouse-anti-phospho(Tyr416)-Src	Merck (05-677)	1:100	
rabbit-anti-CERT	Proteintech (15191-1-AP)	1:1000	
rabbit-anti-STING	Cell Signaling (13647)		1:1000
rabbit-anti-phospho(Ser366)-STING	Cell Signaling (19781)		1:1000
rabbit-anti-IRF3	Cell Signaling (11904)		1:1000
rabbit-anti-phospho(Ser386)-IRF3	Cell Signaling (37829)		1:1000
rabbit-anti-Rab1A	Cell Signaling (13075)		1:1000
rabbit-anti-Rab1B	Abcam (ab116336)		1:1000
rabbit-anti-Rab2A	Proteintech (15420-1-AP)		1:1000
rabbit-anti-Rab2B	Proteintech (11756-1-AP)		1:1000
rabbit-anti-Rab4A	Proteintech (10347-1-AP)		1:1000
rabbit-anti-Rab5A	Cell Signaling (46449)		1:1000
rabbit-anti-Rab8A	Cell Signaling (6975)		1:1000
rabbit-anti-Rab35	Proteintech (11329-2-AP)		1:1000
rabbit-anti-OCRL	Proteintech (17695-1-AP)		1:1000
rabbit-anti-ACAP2	Proteintech (14029-1-AP)		1:10000
rabbit-anti-MICAL1	Proteintech (14818-1-AP)		1:1000
rabbit-anti-MICALL1	Proteinatlas (HPA043480-25)		1:1000
rabbit-anti-FSCN1	Proteintech (14384-1-AP)		1:1000
rabbit-anti-RUSC2	ProSci (155-6659-0.02)		1:1000
rabbit-anti-Slc1	(1)	1:400	1:1000
rabbit-anti-CpoS	(2)		1:200

Table S5 (continued). Primary antibodies used in this study.

Reagent	Source	Dilution used in microscopy	Dilution used in western blot
rabbit-anti-IncA	(3)		1:1000
rabbit-anti-CT222/CTL0475	gift from Ted Hackstadt (RML/NIAID, (4))	1:100	1:1000
rabbit-anti-IPAM	gift from Daniel Rockey (Oregon State University, (5))	1:100-1:200	
mouse-anti-IPAM	gift from Daniel Rockey (Oregon State University, (6))	1:1000	1:1000

References

1. Chen YS, Bastidas RJ, Saka HA, Carpenter VK, Richards KL, Plano GV, Valdivia RH. 2014. The *Chlamydia trachomatis* type III secretion chaperone Slc1 engages multiple early effectors, including TepP, a tyrosine-phosphorylated protein required for the recruitment of CrkI-II to nascent inclusions and innate immune signaling. PLoS Pathog 10:e1003954.
2. Sixt BS, Bastidas RJ, Finethy R, Baxter RM, Carpenter VK, Kroemer G, Coers J, Valdivia RH. 2017. The *Chlamydia trachomatis* inclusion membrane protein CpoS counteracts STING-mediated cellular surveillance and suicide programs. Cell Host Microbe 21:113-121.
3. Cocchiaro JL, Kumar Y, Fischer ER, Hackstadt T, Valdivia RH. 2008. Cytoplasmic lipid droplets are translocated into the lumen of the *Chlamydia trachomatis* parasitophorous vacuole. Proc Natl Acad Sci U S A 105:9379-84.
4. Mital J, Miller NJ, Fischer ER, Hackstadt T. 2010. Specific chlamydial inclusion membrane proteins associate with active Src family kinases in microdomains that interact with the host microtubule network. Cell Microbiol 12:1235-49.
5. Alzhanov DT, Weeks SK, Burnett JR, Rockey DD. 2009. Cytokinesis is blocked in mammalian cells transfected with *Chlamydia trachomatis* gene CT223. BMC Microbiol 9:2.
6. Bannantine JP, Griffiths RS, Viratyosin W, Brown WJ, Rockey DD. 2000. A secondary structure motif predictive of protein localization to the chlamydial inclusion membrane. Cell Microbiol 2:35-47.
